# Mathematical Modeling and Computational Approaches for Pulsed Electric Field Processing in Food Preservation: A Comprehensive Review

**DOI:** 10.3390/foods15010164

**Published:** 2026-01-03

**Authors:** Giovanni Luzi, Khawaja Muhammad Imran Bashir, Wenjing Lyu, Man-Gi Cho, Jae-Suk Choi

**Affiliations:** 1 LSTME Busan, 2B 1276 Jisa-dong, Gangseo-gu, Busan 46742, Republic of Korea; giovanni.luzi@lstme.org (G.L.); man-gi.cho@lstme.org (M.-G.C.); 2 Department of Seafood Science and Technology, The Institute of Marine Industry, Gyeongsang National University, 2-9, Tongyeong 53064, Republic of Korea; imranbashir@gnu.ac.kr; 3 DST Entwicklungszentrum für Schiffstechnik und Transportsysteme e.V., Oststraße 77, 47057 Duisburg, Germany; wenjing.lyu@lstme.org

**Keywords:** pulsed electric fields, food preservation, numerical simulations, non-thermal processing

## Abstract

Pulsed electric field technology possesses a high potential and a bright future in food processing to inactivate microorganisms and reduce enzymatic activity. Processed food shows a higher retention of health-related compounds and an extension of the shelf-life compared to conventional pasteurization methods. This technology is gradually moving from the laboratory and pilot-plant scale to the commercial scale. In the current review, we focus on the way existing knowledge on mathematical modeling and computational approaches is structured, connected, and interpreted across scales. We start with the electroporation models, progressing from those that are derived from simple physical and chemical considerations to those that are based on more complex probabilistic approaches. They attempt to predict how electric pulses create pores in cell membranes and form the basis of kinetic inactivation models. Subsequently, we examine the most common kinetic models of microorganism inactivation, from first-order models to models based on random and probabilistic considerations. We then review the works carried out on the numerical simulations of the electric field in a continuous PEF chamber and the works related to coupled simulations of the electric, fluid flow, temperature, and inactivation kinetic field. Finally, we conclude the manuscript with a section dedicated to the current applications of the PEF process to demonstrate its effectiveness.

## 1. Introduction

**Figure 1:**
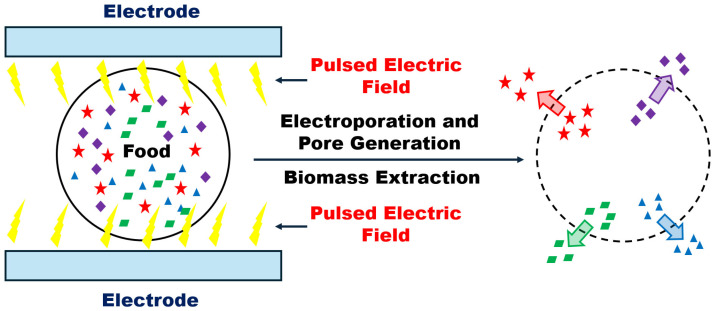
Mechanism of PEF treatment on food products.

In food and bioengineering, the pulsed electric field (PEF) technology has gained much attention in recent years for its ability to preserve, modify, and enhance several products and processes [1,2,3,4]. PEF is a non-thermal processing method that utilizes short bursts of high-voltage electric pulses to treat materials [2,4]; see Figure 1. PEF is widely applied in food preservation and processing due to its ability to extend the shelf life of food while preserving its quality compared to traditional thermal methods [5,6,7,8]. Moreover, PEF facilitates and improves several food processing steps like juice extraction, texture modification, drying, and freezing [9,10,11,12]. PEF also finds applications in the pasteurization of liquid food, and even in wastewater treatment [6,7,8,13]. The lethal effects of PEF on microorganisms have been extensively investigated in the past [8,14,15,16]. In the PEF process, electric field pulses of high intensity (10–80 [kV/cm]) and short duration (1–100 µs) are applied to food products placed in a space maintained between a pair of electrodes. The applied high voltage prevents microbial growth in the food material by electrically breaking down the cell membrane of unwanted microorganisms [1,2,17,18]. This process is called electroporation and leads to the partial or complete damage of microorganisms. The treatment of food using external electric fields causes an increase in the temperature of the food product due to the ohmic heating. Despite this, the temperature of the treated food is usually sublethal in contrast to the extensively utilized thermal treatments, allowing the preservation of the sensory, nutritional, and functional properties of foods [14,15]. Many experimental studies demonstrate that the population of microorganisms in food products can be effectively reduced by using the PEF technique both in batch or continuous mode, and its effectiveness increases if it is combined with a mild temperature increase in the food product [8,16,19,20].

Numerical simulations of the PEF process are often regarded as a valid alternative or at least a complementary approach to expensive or impossible experimental trials. They come into play when experimental techniques are intrusive and disturb the process under investigation [21,22,23,24]. Numerical simulations reduce experimental costs and food waste, permit access to physical variables and the modification of the operational parameters without the need for preparing new samples, grant the process investigation in extreme conditions, and process optimization with the improvement of the design of the PEF machine [21,23,24]. Although experiments are indispensable for validation, numerical simulations provide a cost-effective, efficient, and insightful way to explore PEF mechanisms, optimize parameters, and guide experimental designs [21]. A combination of both approaches often yields the best results.

In this manuscript, we present an integrated, modeling-oriented synthesis of PEF processing, linking electroporation theory, microbial inactivation kinetics, and multiphysics simulations within a unified framework. In this work, we explicitly bridge membrane-scale mechanisms to reactor-scale CFD simulations, assessing model transferability, parameterization challenges, and the feasibility of the numerical implementation. We first describe a PEF setup and illustrate its basic components to provide the reader with a clear understanding of the core technology. This allows for interpreting numerical and experimental results and understanding the technical strengths and limitations of the PEF technology. Afterwards, we summarize the existing electroporation models. They attempt to describe the mechanisms that lead to a cell membrane breakdown under the action of an external electric field, providing a theoretical basis for developing kinetic models. In subsequent sections, we review the kinetic models of microorganism inactivation and the numerical studies of the PEF process available in the literature. As far as the numerical simulations of the PEF process concern, some authors focus only on the simulation of the electric field distribution within the treatment chamber, neglecting the presence of fluid flow, temperature, and inactivation kinetics of microorganisms [2,25,26]. Numerical simulations of the electric field allow for optimizing its distribution in the treatment chamber and for detecting and avoiding isolated peaks. Peaks of the electric field are caused either by the arrangement and the shape of insulators and electrodes or by the presence of unwanted pollutants such as bubbles or globules [2,25]. Other researchers solve the problem completely by coupling the electric, fluid flow, temperature, and inactivation kinetic field [27,28,29,30]. This approach provides the researchers with knowledge about how the fluid flow affects heat conduction, convection, and ohmic heating, and how the growth of microorganisms is influenced by the fluid flow and temperature distribution. The temperature in the treatment chamber should be strictly controlled since it may locally reach values for those where the quality of the food quickly deteriorates [21,24,31]. On the other hand, the knowledge of the velocity distribution of the fluid flow is very important since it determines locally the residence time of microorganisms, affecting their inactivation course [21,24,32]. Therefore, a detailed knowledge of the fluid flow, temperature, electric, and inactivation kinetic distribution is necessary for optimizing the PEF process. Finally, we examine several applications of the PEF treatment process for inactivating microorganisms in foods and discuss how numerical simulations could help to understand and optimize the processes. These are used in food processing industries to check the technological readiness and impact of different potential applications of PEF. The remainder of the manuscript is organized as follows. In Section 2, we describe in detail the types and components of PEF machines typically employed. In Section 3, we summarize the main electroporation models. In Section 4, we revise the main kinetic models for microbial inactivation. In Section 5, we provide the governing equations with the corresponding boundary and initial conditions of a PEF continuous process. They are the mass, momentum, energy, and electrical charge conservation equations. In addition, we also supply a transport equation for the kinetic inactivation of microorganisms. In Section 6, we survey the numerical simulations of the electric field in different PEF chamber configurations, while in Section 7 we summarize the contributions of the coupled numerical simulations of the electric, fluid flow, temperature, and inactivation kinetic field. In Section 8, we discuss some applications of the PEF in food technology to show its capability in industrial processes. Here, we also debate how the use of numerical computations can be employed to improve those processes, and how mathematical and numerical models could be tuned and improved to deliver more reliable predictions. In Section 9, we conclude our manuscript.

## 2. PEF Treatment Devices

### 2.1. Whole System Components

A PEF treatment device typically includes the following items [2,3,4]:

(1) A power supply unit;
(2) Several high-power capacitors;
(3) Switches to release the stored power;
(4) A high-voltage pulse generator;
(5) A treatment chamber for processing food.

**Figure 2:**
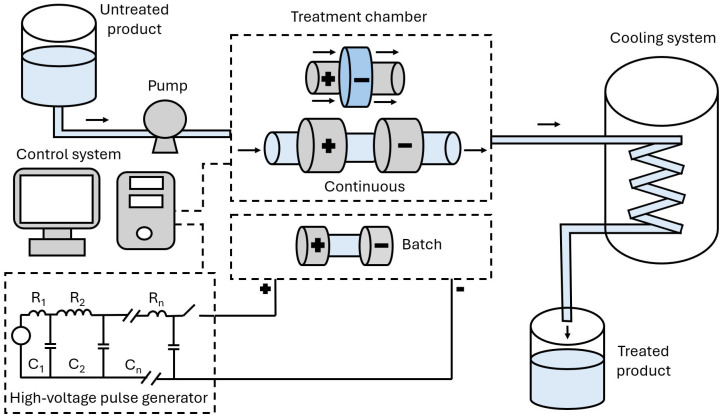
Flow chart of a PEF process.

The power supply unit first conveys high direct current (DC) power from a 110 [V] supply line to a higher voltage alternating current (AC) line [2,3]. This energy is then stored in capacitors before being discharged into the treatment chamber through an electronic switch in fractions of a second. In addition to the essential equipment, additional devices may need to be added to the PEF setup. For continuous processes, a pump is utilized to move the liquid food within the treatment chamber [2,6]. Cooling jackets are often necessary to mitigate the temperature increase in processed food due to ohmic heating [2,4]. An oscilloscope controls the generated pulse waveform and measures the voltage and the current discharged into the treatment chamber [6,33]. Figure 2 illustrates the main components of a typical PEF setup.

### 2.2. Power Supply Unit

A power supply unit of a PEF treatment device provides high-voltage pulses at the required shape, intensity, waveform, and duration [4,34]. It consists of a power supply, energy storage capacitors, and complex control circuits that regulate the duration [4,34], amplitude, and frequency of the pulses. High-voltage power supply units can be classified into two types: the ordinary DC and the capacitor power supply [34,35]. A DC power supply unit converts AC into high-voltage AC and rectifies to a high-voltage DC [35]. On the other hand, the capacitor power supply utilizes as input high-frequency AC in the order of 100 [kHz] to charge capacitors, providing higher repetitive rates than the DC power supply [34]. High-voltage capacitors initially store a large amount of electrical energy that is quickly discharged between electrodes present in the treatment chamber generating short and high-power electric pulses [4,34]. To avoid excessive heating of the electrodes, a circulating cooling system is installed in the PEF treatment device and goes through the capacitor housing absorbing and dissipating the heat during the charging and discharging phases of the capacitors [4]. The capacitance measures the capacitor’s ability to store electrical charges

(1) $$C=\frac{Q}{V}=\frac{\tau }{R}=\frac{\tau \sigma A}{d}$$

where *Q* denotes the charge stored on each plate of the capacitor, *V* is the applied voltage across the plates, τ is the pulse duration, *R* indicates the resistance, σ denotes the conductivity of the processed product, *A* is the whole area of the electrode surface, and *d* represents the total gap between the electrodes. The electrical charge stored in the plates of the capacitor may be written as

(2) $$Q=\frac{1}{2}CV^{2}$$

The peak current can be estimated as

(3) $$I_{max}=\frac{V\sigma A}{d}$$

The maximum temperature increase in the PEF chamber can be computed using thermodynamic considerations

(4) $$\Delta T=\frac{Q}{\rho C_{p}}$$

where in Equation (4), ρ and $C_{p}$ are the density and the specific heat of a liquid food. Equation (4) does not consider cooling effects and holds when the frequency of pulses is very large [34]. Moreover, the pulse frequency may be calculated as

(5) $$f=\frac{n\dot{v}}{V}$$

where *n*, $\dot{v}$, and *V* are the number of pulses, the volumetric flow rate, and the volume of the treatment chamber, respectively.

### 2.3. High-Power Capacitors

High-power capacitors store and rapidly discharge large amounts of electrical energy to generate high-voltage pulses [4,34,35]. They are designed to discharge electrical energy in the nano-to microsecond-scale times, minimizing energy losses and ensuring sharp, high-intensity pulses [34,35]. Capacitors are made of two parallel metal electrodes isolated utilizing material dielectrics such as ceramic, waxed paper, plastic, mica, or liquid gel [12,34]. Since dielectric materials separate the metal electrodes, current does not flow through a capacitor, but a high voltage is generated and stored in the metal plates [4,34]. To sustain high voltages and prevent electrical breakdown, polypropylene, mica, ceramic, plastic films, oil-impregnated paper, and electrolytes are common materials used to fabricate capacitors [12]. Pulse discharge, high-voltage film, ceramic, and oil-immersed capacitors are among the most common types of capacitors employed in PEF technology [34,35].

### 2.4. Switches

The switches are electrical components that quickly connect and disconnect the energy stored in the capacitors to generate a high-voltage pulse to the electrodes [4,34,35]. Two common types of switches are the spark gaps and the solid-state switches. The former controls the electrical discharge utilizing either an ON or ON/OFF mechanism [34,35]. On the one hand, ON switches handle high-voltages and are cheaper than ON/OFF switches. On the other hand, they have a shorter lifespan and operate with lower frequency [35]. Examples of these types of switches include Ignitrons, Thyratrons, Trigatron, and gas spark gap. ON/OFF type switches provide better control of the process of pulse generation, and the capacitors can be partially or completely discharged [34,35]. They utilize high-power semiconductors and provide better control of the pulse characteristics and switching on and off mechanisms [35]. Examples of these types of switches are the gate turn-off (GTO) thyristor, the insulated gate bipolar transistor (IGBT), and the symmetrical gate commutated thyristor (SGCT) [35]. Two important characteristics of the switches are the rise and the fall time. The former affects the speed at which a switch turns on, the pulse shape, and the treatment time. The latter affects the pulse duration, frequency, and speed at which a switch turns off [3,4].

### 2.5. High Voltage Pulse Generator

High-voltage pulse generators (HVPGs) (Table 1) for PEF treatment devices produce short, high-voltage electric pulses that generate a strong electric field in the material that has to be processed to inactivate microorganisms [4,35,36]. HVPGs are extremely important in food processing since the pulse width, voltage, and frequency need to be precisely controlled [3,4,34,36]. HVPGs can generate a wide range of waveforms that can be used in electroporation: exponential, rectangular, multi-pulse, and ramp pulses [4,35,36]. Since the rectangular pulse waveform provides a larger effective area compared to other waveforms, it is the one that is utilized the most. HVPGs can provide either unipolar or bipolar pulses. Bipolar pulses have the advantage of provoking reversing mechanical stresses in the processed microorganisms [4,34,35]. HVPGs can be subdivided into two main groups: classical pulse generators (PGs) and power electronics-based PGs [35,36]. In the case of classical PGs, the energy is first stored in capacitors arranged in parallel and, afterward, is suddenly discharged to other capacitors arranged in series, allowing the generation of the required high-voltage pulses [35]. Examples of classical HVPGs are the Marx generators, pulse-forming network (PFN), Blumlein lines, and magnetic pulse compressor [35]. More recently, due to the advancements of HV semiconductors, HV pulses can be generated utilizing power electronic-based converters [35,36]. Power electronics-based PGs can be subdivided into three groups: non-modular multilevel converter (MMC)-based, MMC-based, and hybrid topologies [35]. Non-MMC-based PGs utilize electronic devices to increase the voltage, then a voltage switch cuts the DC. Examples of non-MMC-based PGs are the solid-state Marx pulse generator (SSMPG), the switched-mode power supply (SMPS), and the capacitor–diode voltage multiplier (CDVM). MMCs are converters connected in series to achieve any required voltage waveform by voltage steps [35]. Examples of MMC-based PGs are the MMC performance context (MMCPC) and the phase-leg MMC based (PLMMCB) PGs. Since non-MMC-based PGs likely need connections of switches in series and MMC-based PGs have limited high-voltage DC input, hybrid PGs are also employed [35]. Two subgroups of hybrid PGs are the non-transformer isolated (NTI) and the transforme-isolated (TI) hybrid PGs [35].

**Table 1:** Main PG technologies used in PEF processing.

Category	Subcategory	Brief Description
PGs	Marx Generator	Charges capacitors in parallel and discharges in series to produce HV pulses
	PFN	A network of capacitors and inductors that store energy and release shaped pulses
	Blumlein Lines	Specialized transmission line configurations that create high-voltage pulses
	magnetic pulse compressor	Devices that reshape electrical pulses utilizing usable saturable inductors
Power electronics-based PGs	Non-MMC-based	Power electronic circuits that generate high-voltage pulses without relying on MMC structures. DC is cut via a switching device.
	MMC-based	Power electronic circuits based on MMC technology. The voltage waveform is built by stacking modules
	Hybrid Topologies	Combine the advantages of MMC and non-MMC technologies, overcoming the limits of voltage input and switching in series

### 2.6. Treatment Chamber

**Figure 3:**
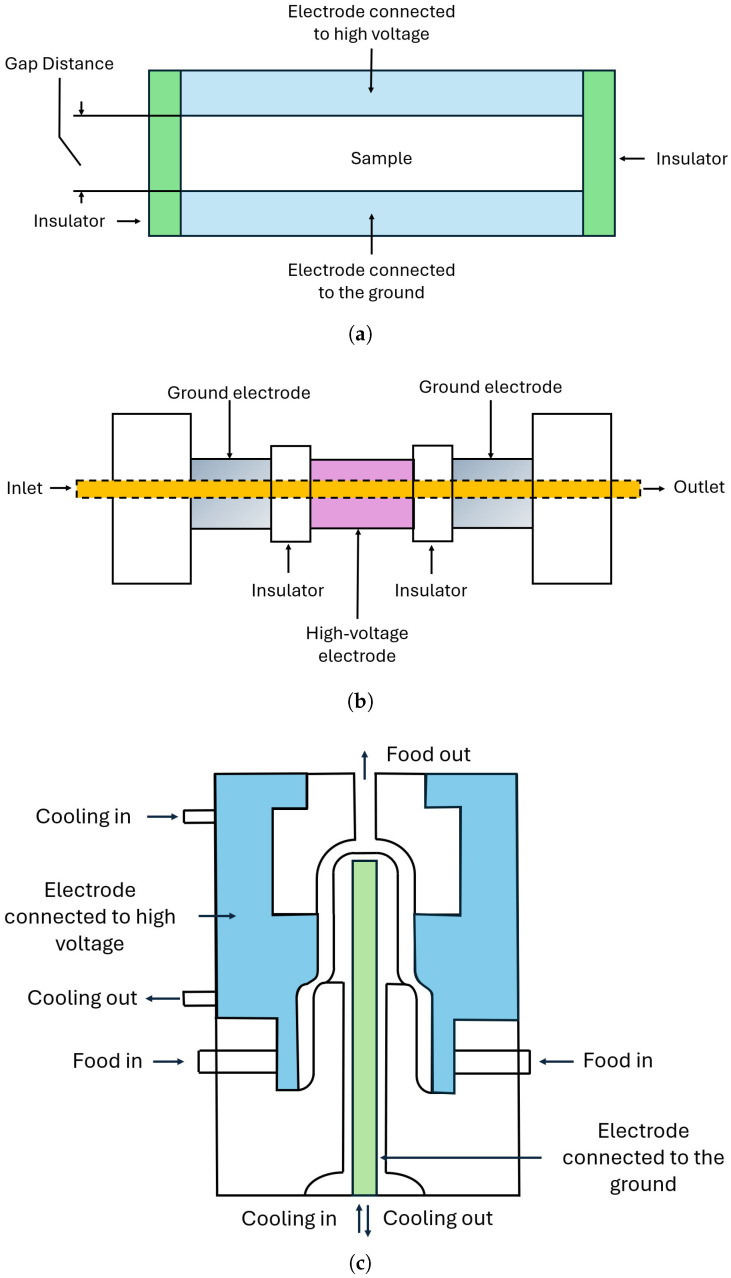
Different types of PEF treatment chambers. (**a**) Static parallel-plate chamber; (**b**) co-field chamber; (**c**) coaxial continuous chamber.

Several laboratory and pilot PEF chambers have been designed and improved over the years [4,24,34,37]. They can be mainly categorized into two types: static/batch chambers and continuous chambers [4]. Batch PEF chambers include parallel-plate and coil chambers, while continuous chambers can be subdivided into co-field, coaxial, and continuous-flow parallel-plate chambers [4,34]. Figure 3 depicts a static parallel-plate (Figure 3a), a co-field (Figure 3b), and a coaxial PEF chamber (Figure 3c). A continuous parallel-plate PEF chamber is analogous to a static one but with longer and wider plates, and a static coil chamber is similar to a parallel-plate one but with the electrodes arranged in a coil configuration [4]. The static parallel-plate treatment chamber consists of two flat parallel electrodes that generate a quite uniform electric field between them. The generated field applies to a food product placed between the electrodes. One of the main limitations of this type of PEF chamber is that large plates are needed for processing a large amount of food. This can be problematic and expensive to implement. Another main limitation is the uneven temperature distribution between the plates [38]. In a coil static electrode chamber, the electrode arrangement in a coiled configuration allows for optimizing the electric field distribution to enhance the process efficiency [34]. However, the coiled electrode design may produce a non-uniform electric field distribution if not optimized and may cause an uneven treatment of processed food [34]. Moreover, like the parallel-plate configuration, this chamber configuration is not suitable for processing a large amount of food. In addition, the overall process efficiency may be affected by the non-optimal electrode configuration and resistance heating [4,34]. A continuous co-field treatment chamber is constructed by adding either two or more parallel plates or coaxial cylindrical electrodes separated by an insulator to form a treatment chamber where the product flows. The co-field configuration ensures a quite uniform electric field, providing that the shape of the insulator is correctly optimized [27,31,32,37]. This type of treatment chamber is in principle ideal for industrial applications with continuous operation since it delivers high throughput. A continuous coaxial treatment chamber consists of two concentric cylindrical electrodes [24]. The inner electrode is made of conductive material and is located along the center axis. The outer electrode is also made of conducting material and surrounds the inner electrode, allowing the liquid food to flow between the two electrodes [24]. The electric field is applied in the radial direction, providing the highest intensity in the vicinity of the inner electrode and gradually decreasing outward [24]. Usually, a dielectric material is placed to separate the two electrodes, prevent short circuits, and improve the uniformity of the electric field. This type of PEF chamber is also ideal for industrial applications with continuous operation systems. The continuous parallel-plate treatment chamber is the analogous continuous version of the static one [34]. Two parallel-plate electrodes are positioned opposite each other and separated by non-conductive electric spacers to maintain a fixed electrode gap. The electric field created between the parallel-plate is quite uniform, and this chamber configuration also is ideal for industrial continuous applications. In the case of laminar flow, strong velocity and temperature gradients may arise provoking uneven distributions [38]. Other types of treatment chambers include the enhanced electric-field continuous chamber [39], the continuous chamber with an ion-conductive membrane [40], and a chamber with electrode reservoir zones [40]. In the enhanced electric field continuous chamber configuration, a conical insulator is employed to remove deposits generated from gas. In the continuous chamber with ion-conductive membranes, conductive ion-permeable membranes are utilized to facilitate electrical conduction. Finally, in the chamber with electrode reservoir zones, dielectric spacer insulators with slot-like openings are employed to enhance the electric field and increase the average residence time.

## 3. Electroporation Models

In this section, we focus on the mathematical and physical models that describe electroporation (Table 2), the key mechanism underlying PEF-induced microbial inactivation. Electroporation models explain how an externally applied electric field destabilizes or perforates the bilayer lipid membrane (BLM), resulting in either irreversible or reversible pore formation. The section progresses from early physical intuitive models to modern, computational-intensive approaches that describe stochasticity and events at the molecular scale. Early electroporation models are based on analogies with electromechanical instability theory and continuum membrane mechanics. Although they all lack stochasticity and molecular details, they provide ready-to use expressions for the critical breakdown voltage, membrane lifetime, and pore nucleation condition. Michael and O’Neill [41] were among the first who investigated the electrodynamic instability of a planar sheet of nonconducting liquid separated by two layers of charged conducting liquids. Although the motion of a membrane cannot be described by the equations of fluid mechanics, several analogies can be found between the stability of a sheet of liquid and the breakdown of a BLM. The model considers that two perturbations of planar surfaces, that is, the symmetric and the antisymmetric waves concerning the mid-plane provoke the instability of the BLM. According to the model, a membrane is unstable due to the perturbation of long-waves, and the breakdown voltage reads

(6) $$V_{c}={\left(\frac{\Gamma h}{2\epsilon_{m}}\right)}^{1/2}$$

where Γ denotes the surface tension coefficient between the layers of conducting and non-conducting liquids. The two main drawbacks of this model are the neglect of the stochasticity of the breakdown process and the dependence of the breakdown voltage on the membrane lifetime. Steinchen et al. [42] and Dimitrov [43] investigated stabilizing and destabilizing mechanical factors and how elasticity, viscosity, and van der Waals forces regulate instability. Steinchen et al. [42] investigated the viscoelastic behavior of a membrane surrounded by two Newtonian viscous fluids performing a linear stability analysis. Specifically, they studied the Kelvin–Voigt and the Maxwell viscoelastic models. On the one hand, the Kelvin–Voigt model is unstable with respect to stretching deformations if the surface tension of the film and the van der Waals forces are negative. Only the shear modulus of elasticity shows a stabilizing effect on the membrane. On the other hand, the Maxwell model is stable with regard to squeezing perturbations as long as the van der Waals forces do not exceed the elastic ones. The elastic shear modulus and the surface elasticity of the membrane have stabilizing effects. Dimitrov [43] devised a viscoelastic film model to compute the breakdown electric potential of lipid bilayers and cell membranes. The model foresees the membrane breakdown in three stages. In the first stage, the fluctuations in the membrane grow, leading to the second stage where molecular rearrangements create discontinuities in the membrane surface. In the last stage, the pores of the membrane expand until the membrane breakdown is completely reached. Performing a linear stability analysis, the authors computed the characteristic time at which the breakdown occurs

(7) $$\tau_{c}={\left(-\frac{G}{\mu }-\frac{\sigma h^{3}k^{4}}{24\mu }+\frac{\epsilon_{m}\epsilon_{o}V^{2}k^{2}}{12\mu }\right)}^{-1}$$

where *G* represents the membrane shear elasticity module, and μ is the membrane shear viscosity. σ, *h*, and *k* are the membrane surface tension, thickness, and the wavenumber, respectively. $\epsilon_{m}$ and $\epsilon_{o}$ denote the membrane relative and the vacuum permittivity. The membrane lifetime may be computed as

(8) $$\tau \approx \left(\frac{\mu }{\frac{\epsilon_{m}^{2}\epsilon_{o}^{2}U^{4}}{24\sigma Gh^{3}}-G}\right)$$

Equation (8) has been subsequently utilized to compute the critical breakdown voltage

(9) $$V_{c}={\left(\frac{24\sigma Gh^{3}}{\epsilon_{m}^{2}\epsilon_{o}^{2}}\right)}^{1/4}$$

Crowley [44] proposed a mechanism where an elastic membrane breaks down due to electrostatic compression. To this end, Crowley [44] modeled the BLM as an electrically insulating elastic layer of material surrounded by two layers of conducting liquids. The two layers of liquid are imagined as a capacitor that has an elastic material between its plates. Performing a balance between the elastic force of the material present between the two layers of liquid and the electric compressive force that develops in the layers of conducting liquid, and assuming a small displacement of the two layers of conducting liquid, Crowley obtained

(10) $$\frac{\Delta }{L}\approx \frac{\epsilon V^{2}}{2EL^{2}}$$

where Δ is the change in thickness, *L* is the initial thickness, and *E* represents the Young’s modulus of the insulating material. *V* denotes the voltage across the capacitor and ϵ the electrical permittivity. In the case of very high-voltages, Crowley devised the relation

(11) $$\frac{\epsilon V^{2}}{2EL^{2}}\approx 0.18$$

which provides the instability criterion as

(12) $$\frac{\epsilon V^{2}}{2EL^{2}}>∼0.18$$

The weakness of this model lies in the hypothesis that the opposite sides of the membrane are flexible and deform freely, yielding a lower voltage value at which the instability may occur.

**Figure 4:**
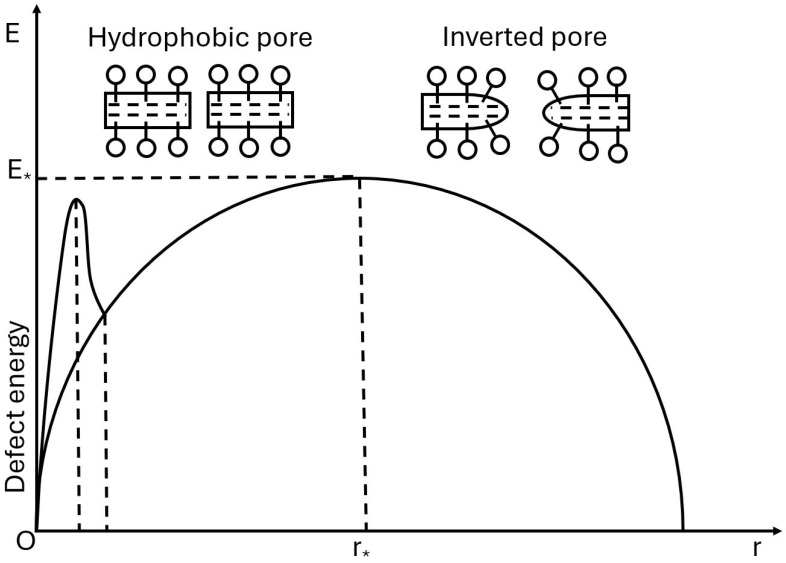
Defect energy against pore radius. The dotted-dashed peak close to the axis of the defect energy indicates the energy barrier needed to maintain hydrophobic pores. Hydrophobic pores that possess a smaller energy barrier close.

Differently, Abidor et al. [45], Pastushenko et al. [46], Weaver and Chizmadzhev [47], and Neu and Krassowska [48] introduced the concepts of pore nucleation and expansion and devised several thermodynamic pore-energy models. A pore experiences several competing forces: linear tension, surface tension, and electric field-induced lowering of the energy barrier. Those models predict a critical pore radius beyond which a pore expands uncontrollably. Additionally, the energy distributions provide insights into the reversible and irreversible electroporation. These models are among the first ones that establish a link between pore energetics, membrane lifetime, and transmembrane voltage, creating a solid theoretical foundation of the modern electroporation theory. Abidor et al. [45] extended the work performed by Crowley by improving the theory of the breakdown of a BLM. They hypothesized that the kinetics of BLM breakdown in the electric field are similar to that for biological membranes. They have in common the onset of conductivity fluctuations before breakdown and the dependence of the membrane lifetime on the magnitude of the potential difference applied. They also assumed that through-going pore defects of different sizes are present in the membranes and that micropores eventually evolve into macropores. They differentiated the pores into hydrophobic and inverted; see Figure 4. The energy required for the formation of a cylindrical through-going pore of radius *r* in the absence of an external electric field is

(13) $$E_{o}=2\pi \gamma r-\pi \sigma r^{2}$$

where γ is the linear tension of the membrane, i.e., the free energy per unit length of the perimeter of a pore, and σ denotes the surface tension of the fluid. In the case of an applied external electric field, Equation (13) modifies as follows:

(14) $$E=2\pi \gamma r-\pi \sigma r^{2}-0.5\pi CV^{2}r^{2}$$

where it is assumed that a pore is small enough so that ions do not penetrate inside it, and the additional electrical energy due to the electric field is modeled as the electrical energy of a capacitor. *V* denotes the voltage applied to the membrane, and *C* is the change of the specific capacitance

(15) $$C=\left(\frac{\epsilon_{s}}{\epsilon_{m}}-1\right)C_{o}$$

where $\epsilon_{s}$ and $\epsilon_{m}$ are the dielectric constants of the water and the membrane, respectively. $C_{o}$ is the specific capacitance of a membrane. The energy dependence on the pore radius reaches a maximum at a critical radius $r^{*}$ (see Figure 4):

(16) $$r^{*}=\frac{\gamma }{\sigma +0.5CV^{2}}$$

where a critical energy level $E^{*}$ of the system is reached

(17) $$E^{*}=\frac{\pi \gamma^{2}}{\sigma +0.5CV^{2}}$$

The membrane pores are stable unless $r^{*}$ is not exceeded. If $r>r^{*}$, the membrane pores expand uncontrollably. For small values of the electric fields, $r^{*}$ remains almost constant. Conversely, for very high values of the electric fields, $r^{*}$ decreases quickly. Pastushenko et al. [46] further developed the model proposed by Abidor et al. [45] and developed a different one to calculate the membrane lifetime both in the case of a membrane with single and multiple defects. To this end, they introduced the function c(r,t) which represents the concentration of defects. It depends on the radius of the defects *r* and the processing time *t* so that c(r,t)dr denotes the probability of an existing defect having a radius between the values *r* and r+dr at the time *t*

(18) $$\int_{0}^{r^{*}}c\left(r,t\right)dr=G_{1}\left(t\right)$$

assuming that the change of pore size is due to thermal motion and due to the action of the forces defined in Abidor’s model and that the electroporation is a random process, Pastushenko et al. [46] formulated the electroporation process utilizing a random walk approach and devising a diffusion-like equation:

(19) $$\frac{\partial c}{\partial t}=D\left(\frac{\partial^{2}c}{\partial r^{2}}+\frac{1}{kT}\frac{\partial c}{\partial r}\frac{dE}{dr}+\frac{1}{kT}\frac{d^{2}E}{dr^{2}}\right)$$

where *D*, *k*, and *T* are the diffusion coefficient, the Boltzmann constant, and the absolute temperature, respectively. The corresponding boundary conditions are

(20) $$\begin{array}{cc} J\left(0,t\right)=0,\;\;\; & \;\;\;c\left(r^{*},t\right)=0 \end{array}$$

where $J\left(r,t\right)$ is the flux of defects, and it is defined as

(21) $$J=-D\left(\frac{\partial c}{\partial r}+\frac{c}{kT}\frac{dE}{dr}\right)$$

By solving Equation (19) with the boundary conditions Equation (20) and performing many algebraic manipulations, Pastushenko et al. [46] obtained explicit formulae for the average lifetime of membranes containing a single defect

(22) $${\bar{t}}_{1}=\frac{{\left(kT\right)}^{3/2}}{4\pi D\gamma {\left(\sigma +0.5CV^{2}\right)}^{1/2}}e^{\left[\frac{\pi \gamma^{2}}{kT\left(\sigma +0.5CV^{2}\right)}\right]}$$

and multiple ones

(23) $${\bar{t}}_{n}=\frac{{\left(kT\right)}^{3/2}}{4\pi cAD\gamma {\left(\sigma +0.5CV^{2}\right)}^{1/2}}e^{\left[\frac{\pi \gamma^{2}}{kT\left(\sigma +0.5CV^{2}\right)}\right]}$$

In Equation (23), *A* is the membrane area. Subsequently, Weaver and Chizmadzhev [47] included in the model the effects of the “spreading resistance” close to the inlet of a pore so that the voltage across a pore $V_{p}$ results in being lower compared to the transmembrane voltage *V*. The variation of the electrical part of the free energy due to the radial displacement of a cylindrical wall of a pore by a value dr may be written as

(24) $$dE_{p}=-\pi rh\epsilon_{w}E_{p}^{2}rdr$$

where $E_{p}=V_{p}/h$ denotes the electric field strength at a pore wall. Equation (24) indicates a voltage drop that can be associated with a “spreading resistance”. It may be written as

(25) $$R_{s}\approx \frac{1}{2\sigma_{e}r}$$

On the other hand, the expression of the internal resistance assumes the form

(26) $$R_{p}=\frac{h}{\pi \sigma_{p}r^{2}}$$

Due to this additional resistance, the expanding force of the electrical field required to enlarge a pore is reduced, and pores expand at a lower rate. In this direction, Parsegian [49] derived several expressions to compute the energy needed by an ion to cross a low dielectric membrane, the pore resistance, and the conduction through pores using a continuum dielectric model. More recently, Joshi et al. [50] derived an expression for the pore formation energy E(r) that includes the dependence on pore population and density. It allows for considering a variable surface tension, incorporates the effects of finite conductivity on the electrostatic correction term, and has a dynamic nature through the dependence on the cell voltage and pore density. The pore formation energy adjusts its value in response to pore formation, avoiding uncontrolled growth or expansion. The model also considers voltage-dependent Born energy corrections originating from the presence of ions in water near pores. The equation for the pore formation energy reads

(27) $$E(r)=2\pi \gamma r-\int_{0}^{r}2\pi \Gamma_{eff}\left(A_{p}\left(r^{*},t\right)\right)r^{*}\;dr^{*}{\left(\frac{C}{4}\right)}^{4}-\pi \left[\frac{\epsilon_{w}-\epsilon_{m}}{h}\right]V^{2}\int_{0}^{r}\alpha^{2}\left(r^{\prime \prime }\right)r^{\prime \prime }\;dr^{\prime \prime }$$

In Equation (27), the third term on the right-hand side represents the electrostatic contribution to the pore formation energy $E_{ES}$. The effective surface tension $\Gamma_{eff}$ in the presence of pores may be written as

(28) $$\Gamma_{eff}\left(A_{p}\right)=\Gamma_{eff}\left(A_{p}=0\right)\frac{1-\frac{A_{0}}{{\left(A-A_{p}\right)}^{2}}}{1-{\left(\frac{A_{0}}{A}\right)}^{2}}$$

where $A_{p}$ is the pore area and $A_{0}$ is the pore area at equilibrium; for more details see [50].

In a different contribution, a slightly modified version of Equation (19) was considered by Neu and Krassowska [48]:

(29) $$\frac{\partial c}{\partial t}=D\left(\frac{\partial^{2}c}{\partial r^{2}}+\frac{1}{kT}\frac{\partial c}{\partial r}\frac{d\phi }{dr}\right)+S\left(r\right)$$

where $S\left(r\right)$ is the source term, which denotes the creation and destruction of the pores. It may be written as

(30) $$S\left(r\right)=\nu_{c}h\frac{\partial E_{nc}}{\partial r}\frac{1}{kT}e^{E_{nc}/kT}-\nu_{d}nH\left(r^{*}-r\right)$$

where $\nu_{c}$ and $\nu_{d}$ are the attempted rate density and the frequency of lipid fluctuation, respectively. *h* denotes the membrane thickness, and $E_{nc}$ is the energy of nonconducting pores at nonzero transmembrane potential. It reads

(31) $$E_{nc}\left(r,t\right)=e\left(r\right)-\pi a_{p}V^{2}r^{2}$$

where $e\left(r\right)$ is the energy of nonconducting pores, and $a_{p}$ is an intrinsic property of the membrane immersed in an aqueous environment. $H\left(r\right)$ is the Heaviside’s function, which accounts for the destruction only of nonconducting pores. The pore energy ϕ is defined as

(32) $$\phi \left(r,t\right)=E\left(r\right)-\pi a_{p}V^{2}r^{2}$$

which considers the presence of an externally applied transmembrane potential

(33) $$a_{p}=\frac{1}{2h}\left(k_{w}-k_{m}\right)\epsilon_{0}$$

where $\epsilon_{0}$ is the vacuum permittivity, and $k_{w}$ and $k_{m}$ are the dielectric constant of the water and of the membrane, respectively. Neu and Krassowska [48] pointed out several drawbacks when one attempts to solve the full Equation (29). First of all, several constants must be known, and it is either challenging or impossible to measure them directly. Second, the variables present in Equation (29) and the quantities that can be measured experimentally cannot be directly linked, and third, an analytical solution of Equation (29) is either very hard or impossible to obtain, and a numerical solution is required. However, even a numerical solution can be problematic to obtain due to the exponential dependence of the creation and destruction rates on the pore energy and due to the presence of very different spatial and temporal scales. To solve this problem, Neu and Krassowska [48] first nondimensionalized (29) to take advantage of the small parameters arising in the resulting equation. Afterward, utilizing asymptotic techniques, they simplified the original partial differential equation (PDE) and reduced it to an ordinary differential equation (ODE)

(34) $$\frac{dN}{dt}=\frac{a}{\mu }e^{-\frac{\phi_{*}}{\epsilon }}\left(1-\frac{N}{\frac{\epsilon^{3/2}}{\mu }be^{-\left(\phi_{m}/\epsilon \right)}}\right)$$

where

(35) $$a=\frac{\left|\phi_{*}^{\prime }\right|}{U_{*}^{\prime }+\left|\phi_{*}^{\prime }\right|}$$

and

(36) $$b=\frac{1}{U_{*}^{\prime }+\left|\phi_{*}^{\prime }\right|}\sqrt{\frac{2\pi }{\phi_{m}^{\prime \prime }}}e^{\int_{1}^{r_{m}}\frac{\phi_{t}-\phi_{m}}{\phi_{r}}dr}$$

and

(37) $$N\left(t\right)=\int_{0}^{\infty }n\left(r,t\right)dr$$

and for more details, see [48]. Equation (34) has several advantages compared to Equation (29). First of all, it is easier to identify terms physically related to electroporation processes. Second, the asymptotic ODE has fewer parameters, and many of them are directly linked to physical quantities that can be measured experimentally. Finally, Equation (34) can be quickly solved numerically with low computational effort and can be easily implemented in CFD codes. Shillcock and Seifert [51] utilized the Monte Carlo method to study how multiple holes behave in a membrane. The pore growth is modeled as a sequential combination of thermal fluctuations, which generate very small holes once an energy barrier is exceeded, and a membrane line tension which regulates the subsequent evolution of the pore size. The main parameters of the model are the energy barrier height, which has to be exceeded to allow pore formation, stretching, and line tension. On the one hand, the effects of the electric field are incorporated into the model by the presence of the energy barrier. On the other hand, the effects of the membrane components are included in the line tension. The pores are treated as two-dimensional circular gas objects that do not interact with each other. Since the energy of a pore radius $r_{j}$ is proportional to its perimeter, a Hamiltonian for a membrane containing *N* pores can be defined as follows:

(38) $$H=\sum_{j=1}^{N}2\pi r_{j}\Lambda$$

where Λ is the line tension. The corresponding partition function is

(39) $$Z\left(T,A,\mu ,\Lambda \right)=\sum_{N=0}^{\infty }e^{\beta \mu N}\sum_{states}e^{-\beta H}$$

where *A* is the area of a membrane, *T* is the temperature, and μ is the chemical potential of a gas pore. The states of the pores are represented by the radii, and pores with equal radii are assumed to be identical. The partition function of the pores is defined as the one of an ideal Maxwell–Boltzmann gas

(40) $$Z\left(T,A,\mu ,\Lambda \right)=\sum_{N=0}^{\infty }e^{\beta \mu N}\sum_{states}\frac{1}{n_{1}!n_{2}!\ldots n_{j}!}e^{-\beta \lambda \sum_{j=0}^{\infty }2\pi r_{j}n_{j}}$$

$n_{j}$ denotes the number of pores with radius $r_{j}$. The grand potential of an ideal gas containing circular pores may be written as

(41) $$\beta \Omega \left(T,A,\mu ,\Lambda \right)=-\frac{1+2\pi \beta \Lambda r_{o}}{{\left(2\pi \beta \Lambda a\right)}^{2}}e^{-\left(2\pi \beta \Lambda r_{o}-\beta \mu \right)}$$

where $r_{o}$ represents the minimum pore radius. Hydrophilic pores can only energetically occur for values of the radii larger than a minimum one. The total average perimeter length of all pores reads

(42) $$\beta \Lambda \langle L\rangle =\frac{\left(1+{\left(1+2\pi \beta \Lambda r_{o}\right)}^{2}\right)}{{\left(2\pi \beta \Lambda a\right)}^{2}}e^{-\left(2\pi \beta \Lambda r_{o}-\beta \mu \right)}$$

while the average number of pores *N* is given by the formula

(43) $$\langle N\rangle =\frac{1+2\pi \beta \Lambda r_{o}}{{\left(2\pi \beta \Lambda a\right)}^{2}}e^{-\left(2\pi \beta \Lambda r_{o}-\beta \mu \right)}$$

In a more recent and detailed approach, molecular dynamics (MD) simulations are used to study electroporation phenomena. MD simulations provide molecular-level insights that are impossible to achieve with continuum models, revealing new molecular-scale mechanisms. However, the high computational demands needed to study the nanoscale and nanosecond regime limit their direct applicability to food processing time-scales. Vernier and Ziegler [52] utilized MD simulations to study the membrane electroporation at the nanosecond and nanometer scale. To this end, they closely investigated the pore-forming lipids and water of a palmitoyl oleoyl phosphatidylcholine bilayer in the case of a minimum porating electric field, which they defined as the smallest external applied electric field necessary to produce one porating event in a 25 [ns] simulation. Their numerical results show that the electroporation process is the result of the alignment of a small number of water and lipid dipoles driven by electric force gradients whose energy barriers are lowered by an external electric field. They also noticed rotations of the head groups of dipoles and water dipole and solvation interactions at the aqueous-lipid interface. Böckmann et al. [53] also utilized MD simulations to investigate the electroporation process of a palmitoyl oleoyl phosphatidycholine bilayer with emphasis on the intermediate stages of the formation process. Böckmann et al. [53] found that asymmetrical variations of the preferred dipole orientation between two monolayers of lipids and water molecules in the presence of external electric fields strongly accelerate the pore formation. Moreover, they proposed a pore formation time based on a statistical theory to compare the numerical outcomes with experiments. The simulations reproduce the same reaction patterns of the different stages provided by experimental data. Qu et al. [54] performed experiments and MD simulations to investigate the electroporation process of myeloma cells. Utilizing also the partial least squares (PLS) regression technique, a machine learning regression method that combines the principal components analysis (PCA) and multiple linear regression (MLR) techniques, they noticed that the number of pores is mainly determined by the pulse strength, while the pore size is dictated by the pulse width. Their results indicate that electroporation events only occur in an electric field whose strength exceeds a certain threshold, and pore formation and size are determined by the difference in the electric potential between the upper and lower cell membranes.

**Table 2:** Main electroporation models used in PEF processing.

Authors	Model Core Idea	Core Mechanism	Key Output
Michael and O’Neill [41]	Electromechanical instability	Long-wave membrane perturbation leads to breakdown	Critical breakdown voltage $V_{c}$
Steinchen et al. [42]	Viscoelastic (Kelvin–Voigt/Maxwell)	Rheological film instability	Stability criteria for viscoelastic membranes
Dimitrov [43]	Viscoelastic disruption	Three-stage membrane destabilization	Characteristic breakdown time and critical breakdown voltage $V_{c}$
Crowley [44]	Electromechanical compression	Electrostatic compressive forces thin the BLM	Instability criterion for membrane breakdown
Abidor et al. [45]	Pore energy	Energy balance for pore formation	Critical energy level and pore radius
Pastushenko et al. [46]	Stochastic random-walk PDE	Diffusion-like equation for the evolution of the defect concentration	Membrane lifetime for single and multiple defects
Weaver and Chizmadzhev [47]	Spreading resistance	Energy balance for pore expansion	Internal pore resistance
Joshi et al. [50]	Stochastic random-walk PDE	Inclusion of several correlations in the pore formation energy equation	New formulation of the pore formation energy
Neu and Krassowska [48]	Pore kinetics are described by an ODE with a few parameters linked to physical quantities	Reduction of the PDE that governs the evolution of defect concentration to an ODE using asymptotic analysis	Time evolution of pore number and size distribution
Shillcock and Seifert [51]	Particle dynamics mesoscopic model	Membrane modeled as coarse-grained particles creating pores under line tension	Dynamics of pore nucleation and growth
Vernier and Ziegler [52]	MD simulations	Alignment of water and lipid dipoles during the electroporation process	Atomic-level mechanism of pore formation and water-defect stabilization
Böckmann et al. [53]	MD simulations	Asymmetrical variation of the dipole orientation accelerates the pore formation	Sequence of pore states and pore formation time
Qu et al. [54]	Hybrid mesoscopic continuum and MD simulations	Pore growth mechanics and MD-informed energetics	Field threshold, pore lifetimes, and large-scale electroporation statistics

It is also worth mentioning that electroporation processes at small scales can also be investigated numerically using conventional numerical techniques. Escoffre et al. [55] and Portet et al. [56] simulated the DNA absorption into chinese hamster ovary (CHO) cells and giant unilamellar vesicles (GUVs). They solved the electric field equations together with an electrodiffusion equation to model the DNA uptake. A comparison between their numerical results with their experimental outcomes of average fluorescence intensity indicates that a good agreement has been obtained. Miklavcic et al. [57] built a three-dimensional finite element model to compute the electric field distribution around and between needle electrodes for an isotropic and homogeneous liver tissue. The electrical field is established for electrochemotherapy and DNA electrotransfer purposes. They validated their numerical results with tests in liver tissue in in vivo conditions. The numerical results permitted the determination of the reversible and irreversible permeabilization threshold, and to utilize voltages and electrode geometries for an optimal exposure of the targeted tissue to the electric field.

The strength of the existing models relies on a sound physical basis for transmembrane potential effects, the ability to predict threshold field values for electroporation, and the flexibility to integrate them with multiphysics models. On the other hand, challenges such as parameter uncertainty, scale separation, biological variability, and pulse diversity remain. For instance, many models require constants that are very difficult or even impossible to determine experimentally, such as line tension, diffusion coefficients, and pore nucleation rates. Additionally, real microorganisms exhibit heterogeneous sizes, shapes, membrane compositions, and intracellular conditions. Moreover, it is challenging to link molecular events at the nano- and microscale to microbial inactivation, which happens on millisecond and micrometer scales. In addition, models are often calibrated to square pulses and do not generalize easily to exponential decay, bipolar, or nanosecond pulses. Electroporation modeling has evolved from early electromechanical instability analysis to sophisticated thermodynamic, stochastic, and molecular approaches. Modern models provide detailed descriptions of pore nucleation, growth, and membrane failure under different electric field conditions, forming the theoretical basis for modern kinetic and numerical simulations of PEF processing. Despite significant progress, challenges remain in accurately parametrizing models for different microbial species, linking microscopic events to microscopic inactivation, and reconciling different temporal and spatial scales. The continued development of multiscale and data-driven models will be crucial for improving predictive accuracy and enabling robust, optimized PEF process design.

## 4. Kinetic Models for Microorganisms

Many kinetic models have been devised to adequately describe microorganism inactivation (Table 3). They provide quantitative and empirical descriptions that allow for the estimation of parameters from experimental data. They also include theoretical considerations that explain how electrical, thermal, and biological factors contribute to microbial death. Those models can be categorized into first-order empirical kinetics, probabilistic and statistical models, and mechanistic-based models. Although each model captures different features of microbial response to the PEF process, there is no single universal formulation since the values of the parameters strongly depend on the medium, microorganism type, pulse characteristics, treatment chamber, and temperature.

### 4.1. First-Order Models

First-order models are among the most common models utilized in the literature. Bigelow [58] proposed a first-order model for heat treatments in the following form:

(44) $$log\left(Y\right)=-\frac{t}{D_{t}}$$

*t* stands for the treatment time, and $D_{t}$ represents the decimal reduction time. The latter is the time required for reducing the number of the surviving microbial population by a factor of ten at a given temperature. The $D_{t}$ value represents a specific thermal resistance of a microorganism [6]. The relation between $D_{t}$ and the temperature *T* is provided by the following equation:

(45) $$log\frac{D_{t1}}{D_{t2}}=\frac{T_{2}-T_{1}}{z}$$

where *z* is the required temperature increase to enhance the lethality of the heat treatment by a factor of 10. The parameters $D_{t}$ and *z* depend on the type of microorganism, the medium, and the previous history of the microorganism [6]. In cases where the survival curves of microorganisms by PEF are linear, Bigelow’s model has proven to be effective. In general, however, first-order models assume that the death of microorganisms occurs due to the inactivation of either a single target molecule or a target location per bacterial cell. They do not consider the lags of death rate at the initial stage of the survival curves and the sublethal injury [59]. Moreover, the behavior of microorganisms and enzymes treated by PEF does not only depend on the treatment time but also depends on different important factors, like the medium, the treatment chamber, the PEF system, the characteristics of the electric pulses, and the treatment temperature [60]. A different first-order kinetics model reads as follows:

(46) $$Y=e^{-kt}$$

where the constant *k* does not depend on the electric field intensity but depends on the temperature according to the Arrhenius’ equation

(47) $$k=k_{T}e^{-\frac{E_{a}}{RT}}$$

where $k_{T}$ represents a rate constant, $E_{a}$ stands for the activation energy, *R* is the universal gas constant, and *T* is the temperature of the medium [61]. The treatment temperature is an important parameter and increases the efficiency of PEF processes, although its effects are complex to estimate [6,62]. In the case of enzyme inactivation, the kinetic constant *k* is usually defined as a function of the electric field strength *E*

(48) $$k=k_{E}e^{\omega E}$$

where the constants $k_{E}$ and ω need to be computed. Another first-order kinetic model may be written as a function of the total electric energy density *Q* as follows [63]:

(49) $$Y=e^{-k_{q}Q}$$

Differently, Levenspiel [64] devised a first-order decimal conversion model for enzyme inactivation that is widely used to compute enzyme inactivation using PEF

(50) $$\frac{Y-Y_{\infty }}{Y_{0}-Y_{\infty }}=e^{-k_{p}P}$$

where $Y_{\infty }$ is the residual enzyme activity after a long-time treatment. *P* includes the effects of the treatment time, pulse frequency, pulse width, and electrical energy density. $k_{p}$ is a rate constant.

Two main advantages of first-order kinetic models are that they can be easily fitted to experimental data and provide a good representation of linear survival curves. However, they fail to capture the shoulder or tailing behavior in survival curves, sublethal injury, and non-linear microbial responses. Due to their strong dependence on experimental conditions, they cannot be considered universal models.

### 4.2. Hülsheger Models

The key idea of the Hülsheger et al. [65] models is that inactivation depends on the treatment time and the electric field strength. Those models also incorporate a critical electric field strength and treatment time. Hülsheger et al. [65] suggested the following model:

(51) $$log\left(Y\right)=-B_{t}\left(t\right)\left(log\left(t\right)-log\left(t_{c}\right)\right)$$

where $B_{t}$ is a regression coefficient and represents the slope of the curve that depends on the treatment time *t*, $t_{c}$ is the critical treatment time for the 100% survival of the microorganisms population. *t* is defined as

(52) t=nτ=nRC

In Equation (52), *R* and *C* are the resistance and the capacitance of the treatment zone. To take into account the effects of the electric fields, Hülsheger et al. [65] proposed a different model:

(53) $$log\left(Y\right)=-B_{E}\left(E-E_{c}\right)$$

where $B_{E}$ is a regression coefficient and represents the slope of the curve that depends on the electric field strength *E*. The critical strength of the electric field $E_{c}$ in the case of a non-conducting sphere in a conducting medium assumes the form [66,67,68]

(54) $$E_{c}=\frac{V_{c}}{1.5a}$$

where $V_{c}$ is the critical membrane potential and *a* represents the mean cell diameter. Equation (54) is only valid for spherical cells and does not hold valid for non-spherical ones. A more sophisticated equation involving both the treatment time *t* and the electric field strength *E* was obtained by Hülsheger et al. [65] by assuming a linear correlation between the logarithm of the survival ratio and the electric field strength, and a linear correlation between the logarithm of the survival ratio and the logarithm of the treatment time:

(55) $$Y={\left(\frac{t}{t_{c}}\right)}^{-\frac{\left(E-E_{c}\right)}{k_{c}}}$$

where $k_{c}$ is a constant. Hülsheger’s models depend on the electric field intensity *E*, the treatment time *t*, or the combination of both. The regression coefficients $B_{t}$ and $B_{E}$ of Equations (51) and (53) vary with different mediums, and larger or smaller values indicate higher or weaker inactivation rates. However, their values do not always increase with decreasing electric field strength or treatment time [69]. The critical electric field strength $E_{c}$ is found to decrease with increasing cell size since the transmembrane potential over the cell is proportional to the cell size [15,70,71]. The constant $k_{c}$ assumes specific values for different organisms. A large value of $k_{c}$ indicates a steep decline of the inactivation curve and a high susceptibility to the PEF treatment, while a small value of $k_{c}$ denotes a larger lifespan of the microorganism and a lower sensitivity to the PEF treatment.

The strengths of the Hülsheger’s models are due to the distinction between subcritical and supercritical electric fields and the cell-size dependency through Schwan’s equation. Their limitations are due to the lack of physical meaning of the regression coefficients, their limited applicability to non-spherical cells, and the variation of the parameters with the medium.

### 4.3. Fermi Model

Peleg [72] proposed the Fermi model which relies on a non-linear sigmoidal equation that combines the effects of the treatment time and the electric field strength

(56) $$Y=\frac{1}{1+e^{\frac{E-E_{c}\left(n\right)}{k_{c}\left(n\right)}}}$$

The model accommodates sigmoid survival curves, which frequently represent the microbial inactivation kinetics. Since the parameters depend on the number of pulses, the model captures the cumulative effects of different pulses. In Equation (56), $k_{c}$ indicates the steepness of the survival curve around the critical electric field strength $E_{c}$. In Fermi’s model, the parameters $E_{c}$ and $k_{c}$ are both exponentially related to the number of applied pulses *n*. A low value of $k_{c}$ indicates a faster inactivation of microorganisms, and an increasing number of pulses suggests a continuous decrease in the value of $E_{c}$ [73]. The steepness parameter $k_{c}$ weakly depends on the treatment time and pulse polarity [74].

The advantages of the Fermi model are that it captures the transition from survival to death of microorganisms and effectively describes threshold phenomena. However, the model is mostly empirical since the main parameters depend on the microorganisms and process conditions. In addition, the model does not directly capture the physics of electroporation.

### 4.4. Log-Logistic Model

Cole et al. [75] proposed a symmetric four-parameter equation based on the distribution of heat sensitivity within the cell population

(57) $$log\left(Y\right)=\alpha +\frac{\omega -\alpha }{1+e^{\left(\frac{4\sigma \left[\lambda -log\left(t\right)\right]}{\omega -\alpha }\right)}}$$

In Equation (57), ω and α are the upper and the lower asymptotes, respectively. σ indicates the maximum slope of the inactivation curve and λ denotes the time at which the maximum slope occurs.

The strengths of the approach are that the model represents the survival curves, incorporating an initial shoulder, the maximum slope at a certain time, and a possible tailing. However, the model may result in over-parametrization for some sets of parameters and requires non-linear fittings.

### 4.5. Giner-Segui

Giner-Seguí et al. [76] devised a kinetic model for the inactivation of enzymes based on two consecutive irreversible first-order steps considering the native, the intermediate active, and the completely inactivated forms at fixed conditions *E*, *f*, and τ:

(58) $$RA=RA_{0}\left[e^{-k_{1}t}-\frac{k_{1}\Lambda }{k_{1}-k_{2}}\left(e^{-k_{1}t}-e^{-k_{2}t}\right)\right]$$

where

(59) $$\Lambda =a_{\lambda }E^{2}+b_{\lambda }E+c_{\lambda }$$

$k_{1}$ and $k_{2}$ are the rate constants of the first and second steps of the inactivation process, respectively. Λ represents the ratio between the activities of the enzyme of the intermediate and native forms. In a subsequent contribution, Giner-Seguí et al. [77] reported that $k_{1}$ is unaffected by the electric field strength *E* and by the polarity of the pulses. On the other hand, $k_{2}$ is affected by the electric field strength *E* and the polarity of the pulses

(60) $$k_{2}=k_{2,a}e^{k_{2,b}E}$$

$k_{2,a}$ and $k_{2,b}$ are the pre-exponential and the exponential parameters.

The main advantage of this model is that by capturing multi-stage inactivation, it is able to distinguish between reversible and irreversible membrane damage. However, since it is mainly designed for enzyme systems, it cannot be straightforwardly applied to predict the inactivation of microbial cells.

### 4.6. Weibull Distribution

A Weibull distribution [78] may be written in the following form [79]:

(61) $$log\left(Y\right)=-bt^{n}$$

The model is simple and describes both downward concave inhibition curves (p>1) and upward concave ones (p<1). If p=1, the model describes a straight line on a logarithmic scale, and it is equivalent to Bigelow’s model. Since the parameter *b* of Equation (61) has no physical meaning and can only have the dimensions of the inverse of time power *p*, Mafart et al. [80] proposed the following modification:

(62) $$Y=e^{-{\left(\frac{t}{\delta }\right)}^{p}}$$

The parameter δ has the dimensions of time, and it is called the time of the first decimal reduction. It is the time needed for the reduction in the surviving microorganisms from $Y_{0}$ to $Y_{0}/10$. The model is highly flexible, as it fits a wide range of microbial responses. However, it presents two major disadvantages. First, a non-linear regression is required to determine the parameters. Secondly, the shape parameters *p* and δ are correlated and not independent, potentially causing a certain instability in estimating the parameters. Moreover, an error in estimating one of the two variables will negatively affect the other one. However, if a single *p* value is chosen, the structural correlation between δ and *p* is eliminated, and the model parameters become independent [81]. δ indicates a resistance of the microorganisms to the inactivation process and decreases with increasing electric field strength *E* [82,83]. It strongly depends on the temperature of the medium and its pH. The condition p>1 indicates that there is a specific time after which the inactivation of microorganisms strongly increases. On the other hand, the condition p<1 denotes that after a specific time, the inactivation of microorganisms by PEF treatment is negligible [59].

### 4.7. Lebovka–Vorobiev Model

Lebovka and Vorobiev [84] proposed an inactivation kinetic model for microorganisms that explicitly incorporates cell size heterogeneity and bilayer electroporation theory. To this end, they assumed a Gaussian distribution of the cell diameters $d_{c}$

(63) $$F\left(d_{c}\right)=\frac{1}{\sqrt{2\pi \Delta }}e^{-\frac{{\left(d_{c}-{\bar{d}}_{c}\right)}^{2}}{2\Delta }}$$

In Equation (63), ${\bar{d}}_{c}$ and Δ indicate the average diameter of microorganisms and the standard deviation. Subsequently, the authors utilized the Monte Carlo method to calculate the lifetime of a cell membrane, using an equation based on the transient aqueous pore model [47]:

(64) $$\tau \left(\theta ,d_{c},E\right)=\tau_{\infty }e^{\frac{\pi \omega^{2}/kT\gamma }{1+{\left(u_{m}\left(\theta ,d_{c},E\right)/u_{0}\right)}^{2}}}$$

$u_{m}$ represents the transmembrane potential of a spherical cell, which can be calculated using Schwan’s equation [85]

(65) $$u_{m}=0.75f\;d_{c}Ecos\left(\theta \right)$$

The parameter *f* depends on the electrophysical and dimensional properties of the surrounding media and the membrane cell, and θ is the angle between the electric field E and the radius r vectors. In Equation (64), *k* is the Boltzmann constant, *T* indicates the absolute temperature, and γ and ω represent the surface and the line tension of the membrane. The voltage parameter $u_{0}$ reads

(66) $$u_{0}=\sqrt{2\gamma /\left(C_{m}\left(\epsilon_{w}/\epsilon_{m}-1\right)\right)}$$

where $C_{m}$ is the specific capacitance of a membrane cell, and $\epsilon_{m}$ and $\epsilon_{w}$ are the relative dielectric permittivities of the membrane and the aqueous phase, respectively. The probability of microorganism damage is estimated using a first-order kinetic equation of the form

(67) $$Y=e^{-\frac{t}{\tau \left(\theta ,d_{c},E\right)}}$$

The surviving fraction of the microorganism is computed using a Weibull distribution.

The advantages of this model are that it combines electroporation physics with survival kinetics and captures cell variability within a population. Nevertheless, it is computationally expensive, being based on the results of Monte Carlo simulations. Moreover, it requires many parameters that are difficult to measure, such as the dielectric constants and the line tension.

### 4.8. Gauss–Eyring Model

The Gauss–Eyring model combines a kinetic and thermal model with exposure time to the PEF treatment and temperature. It may be written as [86]

(68) $$Y=\frac{1}{2}\left[erfc\left(\frac{T-T_{c}\left(t\right)}{\sigma \sqrt{2}}\right)\right]$$

where *T* denotes the PEF process temperature, $T_{c}\left(t\right)$ indicates the temperature corresponding to the inactivation of 50% of the microorganism population, σ represents the width of the temperature distribution, and $erfc\left(x\right)$ is the complementary of the error function. $T_{c}$ assumes the general form

(69) $$T_{c}\left(t\right)=T_{r}-Zlog\left(\frac{t}{\tau }\right)-Z\Delta pH$$

In Equation (69), $T_{r}$ is the reference temperature, *Z* is the temperature rise/reduction required to increase/decrease by a factor of ten the process time, and ΔpH denotes the change in pH. τ is the selected unit of time [5,86].

The model considers temperature, PH, and treatment time, and it is found to be very useful when a PEF process involves moderate heating.

### 4.9. Peleg–Penchina Model

The core idea of the Peleg–Penchina model is to combine together several PEF variables such as the pulse repetition rate, treatment time, electrical conductivity, and electric field intensity into an energy density variable that is linked to the inactivation rate [87]. The energy density may be computed as follows [63]:

(70) $$\Delta E=E^{2}f\tau \sigma R_{t}n_{c}$$

*E* denotes the electric field strength, τ indicates the pulse width, σ is the electrical conductivity, $n_{c}$ represents the number of PEF chambers, and *f* is the pulse frequency. The residence time $R_{t}$ may be estimated as

(71) $$R_{t}=\frac{V}{\dot{Q}}$$

where *V* and $\dot{Q}$ are the volume of the PEF chamber and the volume flow rate, respectively. The inactivation rate reads [63]

(72) $$\frac{dlogS\left(\Delta E\right)}{d\Delta E}=-b\left(\Delta E\right)n\left(\Delta E\right){\left(-\frac{dlogS\left(\Delta E\right)}{b\left(\Delta E\right)}\right)}^{\frac{n\left(\Delta E\right)-1}{n\left(\Delta E\right)}}$$

In Equation (72), $S\left(\Delta E\right)$ indicates the survival fraction, and $n\left(\Delta E\right)$ is a parameter that describes the deviation from the linearity of the inactivation curve. $b\left(\Delta E\right)$ may be written as [63]

(73) $$b\left(\Delta E\right)=log\left(1+e^{k\left(\Delta E-\Delta E_{c}\right)}\right)$$

$\Delta E_{c}$ is a critical value of the energy density at which a significant inactivation begins, and *k* represents the rate at which the inactivation increases when the energy density achieves a critical level.

The main advantage of this model is the combination of electrical and hydrodynamic factors via an energy density variable. However, it requires a detailed knowledge of the process parameters, which are mostly empirical.

### 4.10. Coarse-Graining Model

Wu et al. [88] recently proposed a multiscale coarse-graining method combined with a stochastic probability model to simulate the inactivation of microorganisms during the PEF process. The model considers the effects of field strength, pulse width, treatment time, bacteria concentration, and resistance to inactivation efficacy. The model considers that the membrane breakdown only occurs due to electroporation and neglects other lethal factors such as temperature or chemistry. Bacteria are assumed to be randomly distributed in space, and the distribution is randomly changed every second to simulate a disordered motion. The disruption of a single PEF strike to a microorganism is defined by the accumulated PEF intensity *I*

(74) $$I=E^{2}PW$$

where *E* indicates the electric field strength and PW denotes the pulse width. The striking probability *p* reveals how much the microorganism population is shielded from PEF

(75) $$p=\frac{1}{\sqrt{1+{\left(\frac{PW}{a}\right)}^{2}}}$$

*a* is a parameter similar to a cut-off frequency in a high-pass filtering circuit. The number of surviving microorganisms is computed as

(76) $$n_{i,j}\left(t+1\right)=n_{i,j}\left(0\right)-m_{i,j}\left(t\right)\frac{I}{R}$$

where $n_{i,j}\left(t+1\right)$ is the number of surviving microorganisms during the PEF treatment process at time t+1; $n_{i,j}\left(0\right)$ represents the number of microorganisms before the PEF process; and $m_{i,j}$ is the cumulative striking number of surviving microorganisms at time *t*. It depends on the striking probability *p*, and on the ability of a microorganism membrane to resist the PEF effects *R*. Once the accumulated PEF intensity *I* exceeds the microorganism resistance *R*, the microorganism is considered completely dead.

The two main innovations of this model are that it bridges microscale electroporation effects with macroscale microorganism population dynamics, and it considers spatial heterogeneity and shielding of microorganisms. The two main limitations of this model are the assumption of electroporation as a sole lethal mechanism, and the suitability is only for simulations rather than providing quantitative information about the amount of inactivation.

**Table 3:** Main kinetic models for microorganism inactivation used in PEF processing.

Authors	Model Type	Key Output
Bigelow [58]	Thermal-based first-order model	Predicts a linear inactivation, and it is useful when survival curves have no shoulder or tail
Bendicho et al. [61]	Arrhenius-type first-order model	Predicts a temperature-dependent inactivation rate and captures the thermal contribution occurring during a PEF process
Mendes-Oliveira et al. [63]	Energy-density first-order model	Predicts microorganism survival based on the energy density
Levenspiel [64]	Enzyme-inactivation first-order model	Predicts incomplete inactivation with a plateau
Hülsheger et al. [65], Hülsheger and Niemann [66]	Non-linear field-dependent models	Predicts curvature of survival curves, organism susceptibility, and models non-linear PEF inactivation
Peleg [72]	Sigmoidal (Fermi) model	Strongly predicts the threshold behavior
Cole et al. [75]	Log-logistic model	Predicts survival with the shoulder and tails based on the microorganism’s heat sensitivity distribution
Giner-Seguí et al. [76,77]	Two-step enzyme model	Predicts microbial inactivation in two stages
Weibull [78]	Weibull distribution-based model	Predicts shoulder-tail kinetics, and it is a flexible model with good empirical accuracy
Lebovka and Vorobiev [84]	Stochastic kinetic inactivation model	Predicts the survival of microorganisms based on the probability of membrane damage and a Weibull-type inactivation curve
Mastwijk et al. [86]	Combined PEF-thermal (Gauss–Eyring) model	Predicts the survival curves capturing the PEF effects
Peleg and Penchina [87]	Energy-density ODE-based model	Predicts survival curves based on energy density, flow rate, and includes PEF parameters
Wu et al. [88]	Coarse-grained stochastic model	Predicts the number of surviving microorganisms using a mechanistic model at the microscopic scale

### 4.11. Brief Summary of Kinetic Models for Microorganisms

Many kinetic models are of a strong and empirical nature since they rely on parameter fittings using experimental data. However, this makes them useful for specific systems but limits their generalization across foods, microorganisms, and chamber geometries. All kinetic models lack universality since the inactivation of microbs subjected to PEF treatment depends on the electric field distribution, pulse shape and width, temperature rise, cell-size distribution, physiological state of microorganisms, medium conductivity, and ionic strength. Some authors highlight the importance of population heterogeneity. Models such as Weibull and Lebovka–Vorobiev feature the crucial aspect of biological variability. Novel kinetic models must incorporate the distributions of physical and physiological properties. Models that merge electroporation physics with probabilistic kinetics, such as the Lebovka–Vorobiev model or the coarse-graining model, seem to be the most promising for predictive accuracy. Kinetic models are nowadays being incorporated into numerical simulations. Some models can be straightforwardly incorporated into existing CFD codes, while models based on multiscale simulations remain computationally challenging. However, the trend of kinetic models is toward multiscale, stochastic, and energy-based formulations that consider pore formation mechanics, cell membrane energetics, and energy density concepts.

## 5. PEF Numerical Simulations

### 5.1. Governing Equations

The phenomena occurring in a PEF machine are modeled utilizing the mass, momentum, energy, and electrical charge conservation equations [21]. In addition, a transport equation is employed to model the inactivation kinetics of microorganisms [27]. The continuity equation reads

(77) $$\frac{\partial \rho }{\partial t}+\nabla \cdot \left(\rho u\right)=0$$

where *t* denotes time, ρ is the fluid density, and u represents the velocity vector. The liquid food is usually considered an incompressible single-phase fluid since the concentration of the solid particles is relatively low, the particle size is very small, and the dependence of the density on the temperature is almost negligible. Moreover, the volume fraction of the solid phase is low, the particles barely interact among them, and the Stokes number is also small [89]. Therefore, solid particles are considered passive tracers that do not influence the fluid behavior. Therefore, Equation (77) reduces to

(78) $$\nabla \cdot \left(\rho u\right)=0$$

Another common assumption is to consider the liquid food as a Newtonian fluid, where the dynamic viscosity μ only depends on the fluid temperature *T*. Therefore, the momentum equation reads

(79) $$\rho \left(\frac{\partial u}{\partial t}+\left(u\cdot \nabla \right)u\right)=-\nabla p+\nabla \cdot \left[\left(\mu \left(T\right)+\mu_{T}\left(T\right)\right)\left(\nabla u+{\left(\nabla u\right)}^{T}-\frac{2}{3}I\left(\nabla \cdot u\right)\right)\right]$$

where *p* is the pressure of the fluid flow, $\mu_{T}$ denotes the turbulent viscosity, and *I* is the identity tensor. The left-hand side of Equation (79) comprises the temporal and the convective acceleration, while the terms on the right-hand side of Equation (79) denote the pressure gradient and the divergence of the viscous stress tensor. The energy equation assumes the form

(80) $$\rho C_{p}\left(\frac{\partial T}{\partial t}+\left(u\cdot \nabla \right)T\right)=\nabla \cdot \left[\left(k\left(T\right)+k_{T}\left(T\right)\right)\nabla T\right]+\tau f\sigma E^{2}$$

*E* is the strength of the electric field; $C_{p}$ is the specific heat; *k*, $k_{T}$, and σ are the thermal, turbulent thermal, and electric conductivity; τ denotes the duration of a pulse; and *f* represents the pulse repetition rate. The terms on the left-hand side of Equation (80) denote the temporal and the convective change of the total energy, and the first term on the right-hand side signifies the heat supplied to the fluid by conduction [90]. The last term of Equation (80) describes the Joule heating, i.e., the conversion of electrical to thermal energy. The electric field vector E can be written in terms of the gradient of the electrical potential Φ

(81) E=−∇Φ

assuming that the electric field is irrotational, i.e., ∇×E=0. This implies that no time-varying magnetic field is generated. Assuming an electrostatic field, Ohm’s law for the charge conservation of the electric current can be expressed in terms of Φ as [57]

(82) $$\nabla \cdot \left(\sigma \left(T\right)\nabla \Phi \right)=0$$

The fraction of inactivated microorganisms depends on the specific treatment and on the transport in space and time by the action of the fluid flow. Due to the low concentration of the inactivated cells, their transport by diffusion can be neglected. A transport equation for the activity of inactivated microorganisms can be written as [91].

(83) $$\frac{dF_{p}}{dt}+\nabla \cdot \left(uF_{p}\right)=\pi_{F_{p}}$$

The source term of Equation (83) depends on the treatment temperature and the electric field strength.

#### Turbulence Models

There are many approaches in the literature to model the turbulent viscosity: one- and two-equation turbulence models, large eddy simulation (LES) models, and direct numerical simulations (DNSs). Two-equation models are frequently utilized in turbulence modeling and widely employed in engineering problems. Since they rely on transport equations for the turbulent kinetic energy and length scales, they are in principle complete and do not require any additional information to solve a specific flow problem. However, their derivation is based on specific assumptions that not always hold, i.e., that the turbulent fluctuations are locally isotropic and the turbulent production locally balances the dissipation, meaning that the turbulent scales of the flow are locally proportional to the scales of the mean flow. The eddy viscosity concept is based on this idea since the eddy viscosity represents the proportionality constant between the Reynolds stresses and the mean strain rate. Two popular and widely utilized two-equation models in fluid mechanics are the *k*-ϵ and the *k*-ω turbulence models, for which several modifications exist. In the *k*-ϵ formulation, the equation of the specific turbulent kinetic energy *k* may be written as

(84) $$\frac{\partial \left(\rho k\right)}{\partial t}+\nabla \cdot \left(\rho ku\right)=P_{k}-\epsilon \rho +\nabla \cdot \left[\left(\mu +\frac{\mu_{T}}{\sigma_{k}}\right)\nabla k\right]+P_{kb}$$

The terms on the left-hand side of Equation (84) represent the local and the convective change of the specific turbulent kinetic energy. The first term on the right-hand side denotes the production. It is the specific kinetic energy per unit volume that an eddy acquires per unit of time due to the presence of the strain rate of the mean flow. The second term on the right-hand side of Equation (84) contains the dissipation rate ϵ, which is the mean rate at which the strain rate does work on the viscous stresses. The third term of the right-hand side of Equation (84) denotes the diffusion of the turbulent kinetic energy due to the molecular motion, and the fourth term on the right-hand side of Equation (84) comprises the triple fluctuating velocity correlation and the pressure fluctuations. This term is modeled employing the gradient diffusion hypothesis. The last term on the right-hand side of Equation (84) is the production of the turbulent kinetic energy due to buoyancy [92]. The transport equation for the dissipation ϵ may be written as

(85) $$\frac{\partial \left(\rho \epsilon \right)}{\partial t}+\nabla \cdot \left(\rho \epsilon u\right)=C_{\epsilon 1}\epsilon \frac{P_{k}}{k}-C_{\epsilon 2}\rho \frac{\epsilon^{2}}{k}+\nabla \cdot \left[\left(\mu +\frac{\mu_{T}}{\sigma_{\epsilon }}\right)\nabla \epsilon \right]+C_{\epsilon 1}\frac{\epsilon }{k}P_{\epsilon b}$$

The terms on the left-hand side of Equation (85) represent the rate of change of the dissipation. The first term on the right-hand side of Equation (85) denotes the production of the dissipation, the second term on the right-hand side of Equation (85) is the rate of destruction of the dissipation, the third term describes the spatial redistribution of the dissipation caused by the viscous diffusion, and the fourth one is another form of transport of the dissipation originated by turbulent and pressure–velocity fluctuations. The latter is modeled using the gradient diffusion approach. The last term of Equation (85) represents the buoyancy forces. The turbulent viscosity $\mu_{T}$ reads

(86) $$\mu_{T}=\rho C_{\mu }\frac{k^{2}}{\epsilon }$$

where $C_{\mu }$, $C_{\epsilon 1}$, $C_{\epsilon 2}$, $\sigma_{k}$, and $\sigma_{\epsilon }$ appearing in Equations (84)–(86) are constants calibrated using experiments and DNS data [93].

In the *k*-ω formulation, ω denotes either the rate at which dissipation occurs or the inverse of the time scale at which dissipation occurs. This time scale is determined by the largest eddies. The equations for *k* and ω assume the form [94]

(87) $$\frac{\partial \left(\rho k\right)}{\partial t}+\nabla \cdot \left(\rho ku\right)=P_{k}-\beta_{k}\rho k\omega +\nabla \cdot \left[\left(\mu +\frac{\mu_{T}}{\sigma_{k}}\right)\nabla k\right]+P_{kb}$$

and

(88) $$\frac{\partial \left(\rho \omega \right)}{\partial t}+\nabla \cdot \left(\rho \omega u\right)=\gamma \frac{\omega }{k}P_{k}-\beta_{\omega }\rho \omega^{2}+\nabla \cdot \left[\left(\mu +\frac{\mu_{T}}{\sigma_{\omega }}\right)\nabla \omega \right]+P_{\omega b}+f_{\omega }$$

The turbulent viscosity $\mu_{T}$ for this turbulence model may be written as

(89) $$\mu_{T}=\frac{k}{\omega }$$

Equation (88) considers the same convection, diffusion, production, and destruction processes as (85). The main advantages of the *k*-ω model are its numerical robustness and simple implementation of the boundary conditions since it does not utilize damping functions. Conversely, the main drawback of the *k*-ω formulation is its strong sensitivity to freestream conditions [94].

### 5.2. Boundary Conditions

To solve the system of the governing equations, i.e., Equations (77)–(83), boundary and initial conditions need to be specified on the boundary of the computational domain and at the beginning of the simulations. We review the boundary and initial conditions that need to be applied to a co-field PEF treatment chamber [21,27]. In this configuration, all the electrodes are located in a straight line in the direction of the fluid flow, and two ground electrodes are separated from a central HV electrode by insulators. Since the co-field configuration of the PEF treatment chamber is axisymmetric, the symmetry of the problem could be exploited to reduce the computational problem from a three-dimensional problem to a two-dimensional one [21].

#### 5.2.1. Fluid Flow

The fluid flow in the treatment chamber can be either laminar, when the Reynolds number $Re=\rho \bar{u}D/\mu$ is below 2300 [27], or turbulent [95]. *D* is the diameter of the ground electrode, and $\bar{u}$ is the average value of the axial component of the velocity in a cross-section of the electrode where the fluid flow is fully developed. The boundary conditions for the velocity field at the inlet of the co-linear treatment chamber are either a fixed value or a parabolic distribution of the form $u\left(r\right)=u_{max}\left[1-{\left(r/R\right)}^{2}\right]$, where *r* is the radial coordinate and $u_{max}$ is the maximum value of the velocity. A no-slip boundary condition is imposed at all the walls of the chamber, meaning that the velocity of the fluid flow attains the same value as that of the walls. At the outlet, a zero gradient boundary condition is usually specified [96]. In the case of a two-dimensional domain, a symmetry condition of the form ∂u/∂r=0 is typically employed with w=0, where *w* is the radial component of the velocity field.

At the inlet, a fixed value of the turbulent kinetic energy $k_{in}$ is generally imposed. It depends on the turbulence intensity and the mean value of the inlet velocity. At the outlet, a zero gradient boundary condition n·∇k=0 is specified, and at the solid boundaries, adequate wall functions are set. As far as the boundary conditions for the specific rate of dissipation ω and the rate of dissipation of the turbulence kinetic energy ϵ are concerned, fixed values $\omega_{in}$ and $\epsilon_{in}$ are set at the inlet, and zero gradient boundary conditions n·∇ω=0 and n·∇ϵ=0 are imposed at the outlet. Suitable wall functions for ω and ϵ are specified at the boundaries of the PEF machine.

#### 5.2.2. Temperature Field

At the inlet of the PEF treatment chamber, a constant value of the fluid temperature $T_{in}=T_{amb}$ is set, where $T_{amb}$ is the value of the surrounding ambient temperature. A zero gradient boundary condition, i.e., n·∇T=0 is imposed at the outlet and the insulator boundaries due to the low thermal conductivity of the insulators. At the electrode boundaries, either a zero gradient boundary condition is specified, or a prescribed heat flux boundary condition of the form $n\cdot k\nabla T=\alpha (T-T_{ref})$ is set. In the latter case, the convective heat transfer coefficient α and a reference temperature $T_{ref}$ need to be provided [96].

#### 5.2.3. Electric Potential Field

Although the value of the electrical potential varies with time since the electric field pulsates, a constant value of the electric potential $\Phi =\Phi_{0}$ is usually specified at the boundary of the HV electrode. This choice is motivated because the value of the electrical potential varies considerably between the off-state and the state when the electric field is active. This boundary condition may be difficult to implement and may cause numerical instabilities. To solve this problem, time-invariant potentials are specified at the electrode [22,27,28,31] and the Joule heating source term of the energy equation, Equation (80) is multiplied by the factor fτ. This assumption is valid only if the period between pulses is much smaller than the residence time of a fluid volume in each mesh cell, meaning that each fluid volume undergoes a large number of pulses when it is moving through the PEF chamber. A fixed value of the electric potential Φ=0 is imposed at the ground electrodes, and a zero gradient boundary condition n·∇Φ=0 is set at the insulators, inlet, and outlet of the PEF machine.

#### 5.2.4. Kinetic Model

The initial condition for the fraction of perforated cells in a PEF process is simply F=0. At the inlet, a fixed value F=0 is imposed, and a zero gradient boundary condition n·∇F=0 is set on the insulator, ground, and HV electrodes, and outlet surface.

### 5.3. Fluid Properties

The properties of the liquid food are either determined experimentally or selected from the existing literature. The parameters of Equations (77)–(83) that depend on the temperature are μ, ρ, *k*, $C_{p}$, and σ. If a fluid property is a weak function of the temperature, it can be regarded as a constant and the governing equations often simplify. In the case of water for instance, ρ varies slightly with the temperature in the range between 20 [°C] and 90 [°C] and it can be assumed constant. However, the fluid viscosity μ and the electrical conductivity σ strongly depend on the temperature in this range. On the one hand, μ decreases with an increase in the temperature, affecting the flow field considerably. Conversely, σ increases considerably with an increase in the fluid temperature influencing the strength and the distribution of the electric field. Examples of the dependency of the fluid properties on the temperature are reported and discussed in [23,27,28,37].

## 6. Numerical Simulations of the Electric Field

In this section, we review the works that dealt with the numerical simulations of the electric field alone (Table 4), without considering the presence of the fluid flow and its temperature distribution inside the treatment chamber. The effects of the temperature on the electrical conductivity and the dielectric permittivity of the fluid are disregarded. Numerical simulations of the electric field permit obtaining a detailed knowledge of the strength and distribution of the electric field within the treatment chamber. They allow for isolating the influence of geometry, electrode configuration, and internal heterogeneities on the electric field strength. They are also ideally suited for rapid prototyping, geometry optimization, and identifying spots where the value of the electric field strength may be insufficient for microbial inactivation, because the computational effort required to simulate the electric field distribution is low. Therefore, numerous simulations can be quickly conducted to optimize the PEF chamber geometry. Góngora-Nieto et al. [25] carried out numerical simulations of the electric field distribution within a PEF treatment chamber in the presence of air bubbles. The presence of gas bubbles significantly perturbs the electric field distribution and lowers the voltage at which an electric breakdown of the liquid may occur. The magnitude of the electric field close to the bubble boundaries decreases significantly, and the electric field results more perturbed either when more than one air bubble is present or when bubbles are larger. The authors also scrutinized the cases when two gas bubbles are horizontally and vertically arranged. When two bubbles are vertically arranged, the electric field strength within the bubbles can increase by a factor of approximately two, enhancing the risk of an electrical breakdown with consequent arcing. When the bubbles are horizontally arranged, the electric field within the bubbles also increases but at a lower magnitude, due to the lower permittivity of air compared to the liquid medium. Toepfl et al. [2] conducted two-dimensional simulations of the electric field distribution in the presence of a single and an agglomeration of microorganisms. They showed that the agglomeration of cells and other insulating particles and their orientation may severely reduce the magnitude of the electrical field. Consequently, the critical electrical field strength $E_{c}$ may not be achieved throughout the domain with a consequent reduction of the PEF lethality. Microbial clustering represents a limitation for the PEF process, suggesting that effective mixing and flow-field optimization may be necessary to enhance the process efficiency.

Numerical simulations of the electric field distribution are also employed to optimize the existing types of the PEF chamber geometries. Misaki et al. [26] utilized an improved surface charge method to compute the electric field distribution inside an insulator and to optimize its design by reducing the local peaks of the electric field strength along its surface. Beginning from an initial insulator geometry, the insulator shape is corrected considering the normal Maxwell’s stress and a smooth tangential field strength to obtain an uniform normal field strength in the boundaries of the new geometry. Qin et al. [97] used the finite element method (FEM) to compute the electric field within a parallel plate-electrode chamber and a coaxial continuous chamber. The electric field was initially computed using a known shape of the chamber. Subsequently, the electrode contour was modified to achieve a uniform electric field distribution at the electrode surfaces, ensuring a smooth distribution of the electric field within the whole chambers. The authors designed new PEF chambers based on the results of the numerical computations and conducted experiments utilizing high-voltage exponential decaying and square pulses to verify microbial inactivation. More recently, Mohan et al. [98] conducted experiments and performed numerical simulations of the electric field distribution inside a static chamber. Since generating a uniform electric field distribution using large electrodes is problematic, the authors explored the possibility of replacing large electrodes with several smaller cylindrical ones. Their numerical outcomes, validated with experiments, show that the electric field distribution produced by a 7-electrode pattern is highly uniform. Their contribution shows a practical engineering solution for addressing the issue of the electric field uniformity, which can be adopted in situations where large electrodes are unavoidable.

Numerical simulations of the electric field alone highlight how field uniformity is important to the PEF processing efficacy. On the one hand, localized regions of low electrical field can prevent microorganism inactivation. On the other hand, regions where peaks of the electric field occur increase the risk of arcing. Inclusions, such as bubbles and microbial clusters, strongly perturb the electric field. They behave as insulating obstacles, distorting the electric field, and preventing consistent inactivation. Systematic optimization of the electrode and the insulator shapes leads to a significant improvement in the field uniformity. Since the computational requirements for the simulations of the electric field alone are low, broad parameter sweeps and iterative refinements can be quickly conducted. In summary, numerical simulations of the electric field provide many insights for the design and optimization of PEF treatment chambers. Although they do not consider the interactions between electrical, fluid-flow, temperature, and kinetic inactivation fields, they permit an understanding of how geometrical factors and dielectric heterogeneities affect the treatment uniformity and safety. They also represent the backbone for developing more robust multiphysics models, incorporating hydrodynamic, thermal, and biological components.

**Table 4:** Works on numerical simulations of the electric field within the PEF treatment chamber.

Authors	Focus	Key Findings
Góngora-Nieto et al. [25]	Bubble-induced perturbation of the electric field	Air bubbles lower the breakdown voltage. The vertical bubbles arrangement increases the field strength, with the risk of arcing
Toepfl et al. [2]	Effects of cell agglomeration and orientation	Agglomerates reduce the local field strength, entailing a lower lethality
Misaki et al. [26]	Insulator shape optimization	Reduction of local peaks using modified geometries
Qin et al. [97]	Electrode contour optimization	Chambers with optimized designs yield smoother electric field distributions
Mohan et al. [98]	Electric field uniformity using multi-electrode arrays	A 7-electrode pattern produces a highly uniform electric field

## 7. Coupled Numerical Simulations of the Electric, Fluid Flow, Temperature, and Inactivation Kinetic Field

To design an efficient PEF treatment process, knowledge of the fluid flow and temperature distribution inside the treatment chamber is required. Experimental measurements of the flow and the temperature distribution within the treatment chamber remain a challenging task. The strong electric field generated during PEF treatment affects sensors, provoking malfunctions that may potentially lead to inaccurate readings. PEF treatment often leads to localized and non-uniform heating since the electric field and the conductivity of the treated material vary spatially, impeding accurate and representative temperature readings across the chamber. Moreover, the treatment occurs in very short pulses of the order of micro-seconds, hindering measurements of transient temperature changes in real time. In addition, PEF chambers are compact, and their space limits the insertion and positioning of sensors without disturbing the flow or the electric field distribution. Due to the change in the physical and electrical properties of the fluid during the PEF treatment, it is very challenging to calibrate sensors accurately.

A valid alternative to experimental measurements is represented by numerical simulations. They can provide an accurate representation of the fluid flow, temperature, and electric field distribution within the treatment chamber. To this end, one needs to solve the coupled system of the governing equations, Equations (77)–(83), with suitable boundary conditions. Multiphysics models allow researchers to assess the interplay between fluid flow, temperature, microbial inactivation, and electric field heterogeneities (Table 5). They provide the most realistic representation of PEF processes, allowing process improvement and optimization compared to numerical simulations of the electric field only. However, this comes at the cost of significantly higher numerical effort. Fiala et al. [22] carried out two-dimensional numerical computations of the fluid flow, temperature, and electric field distribution in a co-field treatment chamber. To assess the validity of their computations, they measured the temperature increase in the fluid due to the ohmic heating at specific positions within the chamber, achieving a good agreement between experiments and numerical simulations. In their numerical model, they only considered the dependence of the electrical conductivity on the temperature. Lindgren et al. [31] also conducted two-dimensional simulations to investigate the fluid flow and temperature distributions within four PEF treatment chambers each with a different design shape. Their study highlights how tiny geometrical features can have a high impact on thermal patterns and microbial lethality. They found that geometries with a decreasing insulator diameter provide a more homogeneous temperature distribution without the appearance of "hot spots" and a higher temperature increase in the center of the chamber, increasing the microorganism’s inactivation rate. The center of a PEF chamber is the weakest and the most critical area since the temperature increase achieves a minimum and the velocity of the flow field reaches a maximum value, lowering the efficacy of the PEF treatment. Gerlach et al. [21] also carried out two-dimensional numerical simulations of a co-field treatment chamber, analyzing in detail the fluid flow, temperature, and electric field distribution. They pointed out how a recirculation region close to the walls behind one of the insulators induces a large radial temperature gradient. The portions of the fluid close to the walls recirculate and move at lower velocities compared to those located at the center. Therefore, they are subjected to more electrical pulses with a consequent increase in their temperature. This produces over-treated regions close to the boundaries and under-treated regions in the center of the chamber. Huang et al. [24] performed two-dimensional numerical simulations of fluid flow, temperature, and electric field distribution of three continuous coaxial treatment chambers. Starting with an initial geometry, the shape and the configuration were modified to obtain an enhanced field strength and a homogeneous temperature distribution. Meneses et al. [32] investigated numerically the impact of the insulator geometry and fluid flow conditions. They concluded that the design influences not only the strength and the distribution of the electric field but also the distribution of the velocity field and its maximum value. The authors showed that a suitable insulator shape can promote a more homogeneous electric field distribution and turbulent conditions which enhance mixing effects, improving microbial inactivation. Yan et al. [99] investigated numerically and experimentally a co-field treatment chamber with a particular configuration. It consists of an hourglass-type insulating plate between two parallel electrodes with a spacing between them. The electric field remains concentrated within the insulator hole, where microorganisms are inactivated, and decays towards the electrode surfaces. The authors conducted two-dimensional simulations of the electric field, temperature, and fluid flow distribution of a PEF process where sterilized milk is continuously treated for 1 [h] at 10 [L/h] and preheated at 60 [°C] before entering the PEF chamber. The system achieves the inactivation of the bacteria *Listeria innocua* by a 7-log reduction.

Salengke et al. [100] computed three-dimensional simulations of the fluid flow, temperature, and electric field distribution inside three treatment chambers with different geometrical configurations. In one of the considered geometries, the diameter of the insulating tube is the same as that of the ring electrodes, while in the other two geometries, the diameters of the insulating tubes are smaller than that of the ring electrode. The results of the numerical simulations suggest that the increase in the velocity of the fluid flow due to the diameter reduction strongly diminishes the residence time of a fluid element in the center of the treatment chamber, reducing the efficacy of the PEF treatment process. On the other hand, the diameter reduction provides a more homogeneous and smoother electric field distribution with smaller peaks of the electric field adjacent to the edge of the electrodes. Moreover, their numerical results show that large temperature increases in specific zones of the chamber are lower in turbulent flow conditions compared to laminar ones. Buckow et al. [23] numerically simulated the fluid flow, temperature, and electric field distribution within a co-field chamber for a total of forty-eight (48) process conditions utilizing different settings of voltage *V*, pulse repetition *f*, mass flow rate $\dot{m}$, and pulse length τ. They validated their numerical results by measuring the temperature at five different locations inside the PEF treatment chamber using a fiber-optic system, obtaining a very good agreement between the predicted and the measured values. In a subsequent contribution, Buckow et al. [37] examined how different insulator shapes and sizes affect the distribution of the fluid flow velocity, temperature, and electric field. They validated their numerical output by comparing the calculated effective specific energy input with the one measured experimentally achieving a favorable agreement. Jaeger et al. [95] conducted numerical simulations of the fluid flow, temperature, and electric field distribution in a co-field chamber with the insertion of stainless steel and polypropylene grids. The presence of the grids increases the homogeneity and the strength of the electric field and promotes turbulent flow conditions, which are beneficial for fluid mixing because they avoid high-temperature peaks inside the treatment chamber close to the insulators. In addition, the presence of the grids improves the homogeneity of the temperature distribution of the fluid and leads to enhanced microbial inactivation. Matra et al. [101] investigated the fluid flow, temperature, and electric field distribution within a co-field treatment chamber with elliptic insulator profiles. They specifically investigated how the increase in the treatment units from (1) one to five (5) affects the PEF treatment process performances, revealing that PEF scale-up is a non-trivial task and involves balancing competing physical effects. On the one hand, the increase in the treatment units positively increases the efficiency of microbial inactivation due to the enhanced possibility of microorganisms being inactivated by electroporation. On the other hand, the increase in treatment units with a serial connection globally increases the liquid flow velocity close to the surface of the insulators, thereby reducing the treatment time of the PEF process, lowering its efficacy. Moreover, the temperature of the fluid increases with an increase in the treatment units, suggesting that the number of treatment units and the serial configuration must be judiciously selected. Wang et al. [102] investigated the fluid flow, temperature, and electric field distribution inside a parallel-plate, and coaxial and co-field chambers. The authors proposed a novel structure for the co-field chamber with a parabolic-like shape. This design improves the electric field distribution by significantly reducing isolated temperature peaks and lowering the temperature rise of the fluid flow.

Knappert et al. [27] conducted numerical investigation of the fluid flow, temperature, electric field, and inactivation kinetics distribution of the microalga *Chlorella vulgaris* under PEF treatment in a co-field chamber. They first developed a kinetic model based on a combination of Poisson and Weibull distributions. They validated this model with batch experiments in laboratory electroporation cuvettes and implemented it into the CFD model. The numerical results match reasonably well the experiments in terms of the fraction of the inactivated cells and temperature increase. Schottroff et al. [28] evaluated the performance of two co-field treatment chambers with different inlet configurations. One of the chambers has only one inlet, while the other one has two divided inlets to generate a swirling flow. Computing the flow, temperature, electric, and inactivation kinetic field, the authors showed that the PEF chamber with two divided inlets provides more homogeneous treatment conditions and mixing, resulting in more homogeneous velocity, temperature, and inactivation kinetic distributions with a consequent reduction of temperature and velocity peaks and an increase in the residence time. The authors validated the numerical results with experiments in terms of residence time distribution and temperature increase in the treatment chamber. Although the inactivation of *Microbacterium lacticum* and *ALP* is similar, the chamber with two inlets shows potential for further improvements. Saldaña et al. [29] carried out numerical simulations of the electric field, fluid flow, and temperature distribution within a parallel-plate electrode chamber with tempered electrodes. The results of the numerical simulations reveal that the electric field strength is uniform at different temperatures. The authors compared the results of their numerical simulations with experiments by evaluating the maximum temperature reached in the chamber, achieving a good agreement. The information obtained by the numerical simulations have been also utilized to investigate the inactivation of the microorganism *Salmonella typhimurium* whose inactivation kinetic has been adequately described by a Weibull distribution. Moya et al. [30] developed a numerical model of a lab-scale PEF system to compute the electric field and temperature distribution, microbial inactivation, and treatment time of a solid product after a PEF-ohmic treatment. Their numerical results indicate that the initial temperatures of the electrodes and the solid product are the most important parameters that need to be considered to obtain temperature uniformity in the solid product to improve microorganism inactivation.

Coupled numerical simulations of electric, fluid flow, temperature, and inactivation kinetic field reveal several major key insights. The geometrical features of a PEF chamber are of the utmost importance for the outcomes of a PEF process. Insulator and electrode shape, inlet configuration, and PEF chamber length all directly affect the distribution of all the fields. Additionally, they also determine the degree of flow mixing, temperature uniformity, and residence time within a PEF chamber. Flow structures significantly influence microbial inactivation, since recirculation, high-velocity zones, and low-velocity near wall regions provoke an uneven exposure to electrical pulses, large temperature gradients, and very different microbial inactivation areas. On the one hand, an increase in temperature can be beneficial for microbial inactivation due to thermal effects. On the other hand, an increase in temperature in specific spots of the PEF chamber may be unwanted since it may lead to the deterioration of food properties. Turbulent fluid enhances the uniformity of the treatment process since grids, shaped insulators, and dual inlets reduce temperature peaks and increase microbial inactivation. Residence time distribution also plays a crucial role since an insufficient residence time leads to under-treated fluid zones even with uniform electric fields.

**Table 5:** Works on coupled numerical simulations of the electric, fluid flow, temperature, and inactivation kinetic field within the PEF treatment chamber.

Author	Coupled Numerical Model	Key Findings
Fiala et al. [22]	Electric, fluid flow, and temperature field	The dependence of the electrical conductivity on the temperature significantly affects the electric field distribution
Lindgren et al. [31]	Electric, fluid flow, and temperature field	Chambers with decreasing insulator diameter produce a more homogeneous temperature distribution and reduce the presence of hot spots
Gerlach et al. [21]	Electric, fluid flow, and temperature field	Wall recirculation behind insulators provokes large radial temperature gradients
Huang et al. [24]	Electric, fluid flow, and temperature field	Modified coaxial geometries enhance the electrical field strength and homogenize the temperature distribution
Meneses et al. [32]	Electric, fluid flow, and temperature field	Insulator shapes strongly affect the electric and fluid flow fields, promoting turbulence and mixing
Yan et al. [99]	Electric, fluid flow, and temperature field	An hourglass insulator confines the electric field and permits a 7-log reduction of *Listeria innocua* in a continuous treatment chamber
Salengke et al. [100]	Electric, fluid flow, and temperature field	The insulator diameter significantly affects the flow velocity, residence time, and electric field uniformity. Turbulent flow reduces temperature peaks
Buckow et al. [23]	Electric, fluid flow, and temperature field	48 process conditions simulated and validated via fiber-optic measurements
Buckow et al. [37]	Electric, fluid flow, and temperature field	The insulator shape significantly influences the fluid flow velocity, temperature, and electric field
Jaeger et al. [95]	Electric, fluid flow, and temperature field	Stainless-steel and polypropylene grids homogenize the electric field, induce turbulent flow conditions, reduce temperature peaks, and improve mixing and inactivation
Matra et al. [101]	Electric, fluid flow, and temperature field	The number of PEF units connected in series must be carefully selected
Wang et al. [102]	Electric, fluid flow, and temperature field	A novel PEF chamber design reduces isolated temperature peaks and improves electric field uniformity
Knappert et al. [27]	Electric, fluid flow, temperature, and inactivation kinetics field	A coupled CFD-kinetic model delivers good predictions of microbial inactivation
Schottroff et al. [28]	Electric, fluid flow, temperature, and inactivation kinetics field	A dual inlet chamber creates a swirling flow that improves the homogenization of the fluid flow, temperature, and inactivation kinetic field
Saldaña et al. [29]	Electric, fluid flow, temperature, and inactivation kinetics field	Tempered electrodes improve the homogenization of the electric field
Moya et al. [30]	Electric, fluid flow, temperature, and inactivation kinetics field	The electrode and product initial temperature strongly affect the microbial inactivation effectiveness

## 8. Current Applications and Development of PEF

PEF technology has been widely used to inactivate microorganisms in a wide variety of foods. Several works demonstrate that both the process temperature and the electric field intensity strongly influence the inactivation efficacy. Additionally, foods matrix composition also play a significant role. In the following subsections, we summarize several applications of PEF for inactivating microorganisms in food products and offer critical insights concerning how numerical simulations could improve the process understanding and efficiency.

### 8.1. PEF Inactivation in Fruit-Based Products

Several contributions have investigated PEF effectiveness in juices, purées, and fruit derivatives.

Geveke et al. [7] investigated the inactivation of *Escherichia coli (ATCC 35218)* inoculated on buffered water peptone (BWP) and fresh strawberry purée. On the one hand, Geveke et al. [7] set the electric field strength between 28.0 [kV/cm] and 33.6 [kV/cm] and the outlet temperature of the PEF treatment chambers between 55.0 [°C] and 57.5 ± 0.5 [°C] to treat the BPW. On the other hand, they selected an electric field strength of 24.0 [kV/cm] and chamber outlet temperatures between 45.0 [°C] and 52.5 ± 0.5 [°C] to treat strawberry purée. The population of *Escherichia coli* decreased by 6.5 log in BPW at E= 30 [kV/cm] and at 57.5 [°C], and 7.3 log in strawberry purée at E= 24 [kV/cm] at 52.5 [°C], proving that a significant change of the applied electrical strength led to minimal difference of the inactivated organisms. However, a small increase in the value of the outlet temperature of the PEF treatment chamber led to a significant increase in the amount of inactivated microorganisms. After the PEF treatment, the strawberry purée showed a bright red color that remained good for approximately three months. In their study, the authors also developed a continuous high-data acquisition system that ran on a desktop computer and could record a large amount of pulses for validation purposes. Plaza et al. [103] evaluated the effects of high-pressure (HP), PEF, and low-pasteurization (LP) treatments on carotenoid and flavanone content of freshly squeezed orange juice during refrigerated storage. The HP treatment was conducted at p= 400 [MPa] and T= 40 [°C], for approximately 60 [s] and the PEF treatment was conducted at E= 35 [kV/cm] for 750 [µs], and the LP treatment was conducted at T= 70 [°C] for approximately 30 [s]. Immediately after the treatment, the HP-treated orange juice showed an increase in the extraction of carotenoid content of 45.19% and of flavanone content of 15.46%. Moreover, the authors also reported an increase of 30.89% of vitamin A value compared to the case of untreated juice. On the other hand, the PEF and LP-treated orange juices did not show any significant change in the carotenoid and flavanone content and vitamin A value in comparison with the untreated juice. The total carotenoid and vitamin content remained higher after 40 days of refrigerated storage at T= 4 [°C] after the HP treatment compared to the PEF and LP ones, while the total flavanone content remained higher after 20 days of refrigerated storage at T= 4 [°C] after the HP treatment. Mosqueda-Melgar et al. [104] applied the PEF treatment on *Salmonella enteridis*, *Escherichia coli*, and *Listeria monocytogenes* inoculated in melon and watermelon juices. The electric field strength is 35 [kV/cm], and the pulse duration is 4 [µs]. The authors achieved a maximum inactivation of 3.75 log of *Salmonella Enteridis* in melon and watermelon juices using treatment times of 1250 [µs] and 2000 [µs], respectively. With regards to *Escherichia coli*, the population reduced by 3.91 log and 4.01 log in melon and watermelon juices employing 1250 [µs] and 2000 [µs], respectively. Regarding the reduction in *Listeria monocytogenes* concerns, the microbial population was reduced by 4.27 log and 3.77 log in melon and watermelon juices using a treatment time of 2000 [µs]. The process temperature never exceeded 40 [°C] at the outlet of the treatment chamber. Zhao et al. [105] studied the effects of PEF with regards to the inactivation of *Escherichia coli* and *Staphylococcus aureus* in green tea, its color after the treatment, green tea polyphenols (GTP) content, and free amino acids. The authors utilized three different electric field strengths, 18.1 [kV/cm], 27.4 [kV/cm], and 38.4 [kV/cm], four total treatment times in the case of *Escherichia coli*, 40 [µs], 80 [µs], 120 [µs], and 160 [µs], and five total treatment times in the case of *Staphylococcus aureus*, i.e., 40 [µs], 80 [µs], 120 [µs], 160 [µs], and 200 [µs]. In the case of *Escherichia coli*, Zhao et al. [105] reported a log reduction of 2.2, 3.3, and 5.6 after 160 [µs] for each of the three electric field strengths used. In the case of *Staphylococcus aureus*, the inactivation attained a 4.9 log reduction after 200 [µs]. Moreover, the color, GTP, and total free amino acids did not change significantly after the PEF treatment. Huang et al. [106] evaluated the PEF resistance of three strains of microorganisms, *Staphylococcus aureus*, *Escherichia coli*, and *Saccharomyces cerevisiae* in grape juice at different electric field strengths and an initial temperature of 40 [°C]. On the one hand, the authors reported a maximum of 6.09 log inactivation using a treatment time of 45 [µs] and an electric field strength of 27 [kV/cm] in the case of *Saccharomyces cerevisiae*, reaching a maximum process temperature of 41.5 [°C]. On the other hand, the authors obtained maximum inactivations of 3.36 log and 2.27 log in the case of *Staphylococcus aureus* and *Escherichia coli*, respectively, employing a treatment time of 275 [µs] and the same electric field strength, with a maximum process temperature of 48.8 [°C]. In addition, Huang et al. [106] employed the Weibull model to describe the inactivation of microorganisms utilizing the treatment time and the specific energy as control parameters.

The application of PEF to fruit-based products allows pigments, vitamins, and sensory attributes to be largely preserved compared to conventional thermal treatments. However, these systems are not homogeneous since electrical conductivity, viscosity, particle size distribution, and phase composition, i.e., pulp, fibers, and air inclusions vary, leading to heterogeneous electric field and temperature distributions inside the treatment chamber. These inhomogeneities can create zones where the microbial inactivation is less efficient, contributing to the higher resistance of some microorganisms in certain juices. In this regard, numerical simulations may play a central role in future PEF process optimization. Multiphysics models that simultaneously resolve the field distributions of electric, fluid flow, temperature, and microbial inactivation kinetics can provide spatial and temporal information on lethal and sublethal areas inside PEF processing chambers. Considering also the dependency of the conductivity on the temperature and non-Newtonian rheology, multiphysics models can help to identify optimal operating conditions to minimize field heterogeneities while avoiding excessive temperature increase. Combined CFD-ML models could be used to optimize electrode shape and arrangement, pulse parameters, and flow conditions for different fruit-based products.

### 8.2. PEF for Dairy Products and High-Protein Systems

PEF is also utilized to inactivate microorganisms in dairy applications, but the presence of proteins can influence the electroporation behavior and the conductivity of the food matrix. Schottroff et al. [107] investigated the inactivation of *Listeria innocua* in the pasteurization of whey protein formulations utilizing the PEF technique. To this end, they studied the influence of several process parameters such as the inlet temperature, the PEF electric field intensity, the protein content, and the pH on the inactivation process conducted in a continuous co-field treatment chamber. The whey protein isolate (WPI) was increased from 2 % [*w*/*w*] up to 10 % [*w*/*w*], and the inlet temperature ranged between 20.0 [°C] and 40.0 [°C], which resulted in outlet temperatures varying between 37.0 [°C] and 58.0 [°C]. The pH values tested were 4 and 7. The authors reported a maximum inactivation of 6.51 log at 2 % [*w*/*w*] WPI, inlet temperature equal to 20.0 [°C], and pH equal to 4 without significant damage of the nutritional components compared to the situation before applying the PEF treatment. Wu et al. [108] studied the protein oxidation, colloidal properties, and electrophoresis patterns of egg white solution subjected to PEF at E= 25 [kV/cm] for different treatment times varying from 400 [µs] up to 800 [µs]. They found that the protein solubility and protein content declined as the treatment time steadily increased, the particle size distribution (PSD) significantly changed if the treatment time t= 400 [µs] was exceeded, and the Z-average size increased with increasing treatment time. Nevertheless, protein oxidation was not detected although sulfhydyl groups increased and protein aggregates formed as the treatment time reached t= 800 [µs]. Sepulveda et al. [109] investigated the combined effects of PEF with mild thermal treatment to extend the shelf life of whole milk. The applied electric field is *E* = 35 [kV/cm] with a pulse width equal to 2.3 [µs]. The whole milk was heated up to 65 [°C] for 10 [s]. The combined treatment extended the shelf life of the whole milk for a minimum of 24 [days], showing superior performance compared to the thermal treatment alone and to a combined PEF and thermal treatment performed at lower temperatures [97]. This experimental investigation also highlights that the PEF treatment effectively killed enteric bacteria while it less effectively eradicated psychrotropic and mesophilic bacteria compared to a high-temperature short-time (HTST) pasteurization method.

The presence of complex and strongly coupled food matrix interactions appears as PEF are applied to dairy and protein-rich food products. Protein denaturation, aggregation, and conformational changes modify the nutritional and functional properties of the food product. Additionally, its dielectric behavior, electrical conductivity, and rheology are altered during the process. These changes of the food properties affect the PEF process itself since the strength of the electric field, the temperature, and the residence time distributions also vary accordingly. Therefore, microbial inactivation, protein stability, and preservation of food quality cannot be considered independently, especially in concentrated protein systems such as whey formulations, egg white solutions, and whole milk. Future research will increasingly rely on numerical simulations since they capture multiphysics and multiscale interactions. Multiphysics models that integrate temperature-dependent protein denaturation and aggregation mechanisms could offer a clear understanding of how the variation of PEF parameters affects both microbial lethality and protein structure. Additionally, considering colloidal-scale descriptions, i.e., population balance models for aggregate growth or effective-medium approaches for variation of the conductivity, would allow simulations to incorporate more realistic electrical and thermal properties changes occurring during the PEF treatment. Numerical simulations could also be utilized to identify operating conditions that maximize microbial inactivation while remaining below critical thresholds for irreversible protein aggregation or an excessive increase in viscosity. In the future, numerical computations may enable tailored matrix-specific PEF strategies, where PEF parameters and treatment chamber designs are customized to obtain the exact amount of protein concentration and pH. This rational, model-based design optimization of PEF processes for dairy and protein-rich foods will ensure both safety and high product quality, also at an industrial scale.

### 8.3. PEF Effects on Vegetables, Starches, and Plant Tissues

Beyond microbial inactivation, PEF process enhances mass transfer, texture, and processing efficiency in plant-based foods. Fauster et al. [9] studied the influence of PEF pretreatment on the production of industrial french fries, with emphasis on potato texture and several quality aspects, like feathering, peeling, breaking, oil uptake, and starch loss among others. They experimentally found that PEF treatment reduces the proportion of feathering by approximately 40% by applying an energy input of 0.2 [kJ/kg], and by 80% by applying an energy input of 1.0 [kJ/kg]. Starch loss decreased from 7.1 [kg/ton] to 5.0 [kg/ton], breaking loss reduced from 11% to 6% regardless of the specific energy input, and fat uptake reduced from an average of 7.5% to 6.8% regardless of the increased energy input from 0.2 [kJ/kg] to 1.0 [kJ/kg]. Moreover, increasing the energy input from 0.2 [kJ/kg] to 1.7 [kJ/kg] significantly affects the lump proportions. However, the PEF treatment does not show any influence on the steam peeling behavior of potatoes or in the extrusion work of by-products such as purée. Bai et al. [10] investigated how the PEF pretreatment influence the structure, thermal properties, texture, starch hydrolysis, and aroma of cooked rice. To this end, they focused on two treatment conditions T1 and T2. In the case of T1, V= 6 [kV], f= 200 [Hz], and t= 6 [µs] and t= 120 [s], and in the case of T2 V= 18 [kV], f= 1000 [Hz], and t= 6 [µs] and t= 30 [s]. The starch hydrolysis increased in both PEF treatment cases compared to the untreated cooked rice, while the cooked rice that underwent the treatment T1 showed larger pores and the formation of irregular arrays compared to the untreated case. The rice that underwent the treatment T2 shows a looser structure with a small change in pore size. Moreover, the hardness, cohesiveness, springiness, gumminess, and chewiness of rice that experienced the T1 and T2 PEF pretreatments decreased compared to the untreated rice. The crystallinity of rice that underwent the PEF pretreatments also decreased. Andreou et al. [11] assessed the potential of PEF with regard to the increase in productivity, improvement of the quality, and valorization of waste in the tomato food industry. The authors applied the PEF pretreatment in three different steps. In the first step, they treated unpeeled tomatoes utilizing an electric field E= 0.5–1.5 [kV/cm] and the number of pulses varied between 0–8000. As a result of the PEF treatment, the work needed to detach the peel from tomatoes decreased up to 72.3%. In the second step, chopped tomatoes were treated before juicing at E= 0.5–2.5 [kV/cm] for 0–4000 pulses, enhancing the overall yield up to 90.2%. In the last step, the PEF treatment was applied to tomato waste at E= 1.0–5.0 [kV/cm] for 0–5000 pulses. The extraction of high-value compounds such as carotenoid and lycopene, and the extraction of phenolic compounds increased significantly. The pulse width and frequency were held fixed in all the experimental steps at 15 [µs] and 20 [Hz]. Cheng et al. [110] investigated how PEF affects the quality and structure of duck eggs during pickling. To this end, the authors also utilized several machine-learning-based models, i.e., extreme gradient boosting (XGB), support vector regression (SVR), artificial neural network (ANN), and genetic algorithm (GA)-ANN, to predict the salt content. Their experimental results after 20 days indicate that the salt content increased by 21.6% at 3 [kV/cm], the egg white and yolk moisture content decreased by 2.07% and 12.4%, respectively, the yolk index and the oil content raised by 10.3% and 39.8%, respectively, the hardness of egg whites and yolks incremented by 27.7% and 24.2%, respectively, and the springiness increased by 7.9% at 2.5 [kV/cm]. In addition, the free sulfhydryl groups and the surface hydrophobicity rose by 6.5% and 35.4% after 20 and 10 days, respectively, at 2.5 [kV/cm]. Finally, their scanning electron microscope pictures elucidate that all the treated samples present many voids in their surfaces compared to untreated ones.

PEF-induced softening and permeability increase in plant tissues derive from a complex interaction between membrane permeabilization, mechanical weakening of cell walls, and fluid redistribution inside the porous cellular matrix. On the one hand, electroporation models describe well the pore formation at the membrane scale. On the other hand, they fail to capture the macroscopic textural changes observed in plant-based foods after the application of a PEF process, such as reduced hardness, enhanced mass transfer, and increased porosity. The plant tissues’ behavior under PEF treatment can be described by a hydrated and deformable porous media, where cells, intracellular spaces, and cell walls produce a mechanical response to electrically induced stresses. PEF treatment not only modifies the membrane permeability but also causes local pressure gradients, cell wall deformations, and irreversible structural rearrangements, which govern tissue softening and transport enhancement. Numerical simulations offer a valid framework to understand the gap between electroporation phenomena at the microscale and macroscopic food quality attributes. Multiphysics models can explicitly consider the interaction between the transmembrane potential, local osmotic pressure, cell turgor loss, and mechanical relaxation of the tissue matrix. They predict the changes of permeability, local collapse or expansion of cellular structures, moisture and oil distribution, and effects of solute transport. Although these phenomena are consistently reported experimentally, they remain poorly quantified. Multiphysics simulations could significantly improve PEF design and control, allowing systematic investigations of how PEF parameters influence the mechanical properties of tissues, the maximization of mass transfer, and the minimization of undesirable quality losses. For example, numerical computations could identify treatment conditions where the increase in membrane permeability is achieved without excessive structural damage, starch loss, or oil uptake. However, this is challenging due to the complexity of the plant tissue and the lack of experimentally validated material parameters.

### 8.4. PEF Inactivation in Alcoholic Beverages and Low-pH Systems

PEF also finds applications to beverages where the presence of alcohol and low PH lowers the efficacy of the process. Milani et al. [111] applied the PEF treatment to inactivate *Saccharomyces cerevisiae* ascospores in nine different beers with different alcohol content. To this end, they utilized an electric field strength of 45 [kV/cm] with 46 pulses of 70 [µs] each. In the first trials, the authors kept the experimental temperature below 43 [°C], achieving log reductions of 0.2% and 2.2% for alcohol concentrations of 0 [alc/vol] and 7 [alc/vol], respectively. Afterward, Milani et al. [111] used the PEF process with a process temperature of 53 [°C] to inactivate *Saccharomyces cerevisiae* in beers with alcohol concentrations of 0 [alc/vol], 4 [alc/vol], and 7 [alc/vol], obtaining additional 0.7, 2.1, and 1.8 log reductions, respectively. Geveke and Kozempel [112] inoculated the yeast cells *Saccharomyces cerevisiae* and *Candida stellata*, and the bacterial cells *Escherichia coli* and *Listeria innocua*, in sterilized deionized water and subjected them to PEF of 12.5 [kV/cm] intensity at 2, 5, 10, and 20 pulses. *Saccharomyces cerevisiae* and *Candida stellata* were diminished approximately by 3.3 log [cfu/mL] and 3.5 log [cfu/mL], respectively, with five pulses. The population of *Escherichia coli* was reduced approximately by 1.3 log [cfu/mL] with 20 pulses, and the population of *Listeria innocua* was lowered by 1 log [cfu/mL] and 3 log [cfu/mL] at pH = 6.6. and pH = 3.8. Mendes-Oliveira et al. [63] utilized the PEF treatment to inactivate *Escherichia coli* and *Salmonella typhimurium* inoculated in apple cider. To this end, they tested combinations of several electric field strengths, 20 [kV/cm], 25 [kV/cm], 30 [kV/cm], and 35 [kV/cm] at different repetition rates, 500 [pps], 750 [pps], 1000 [pps], 1250 [pps], and 1500 [pps] to reduce the bacterial population up to 5 log [cfu/mL]. The combinations 1250 [pps] and 1500 [pps] at 35 [kV/cm] were excluded since the authors already achieved a log reduction of 5 log [cfu/mL] at 30 [kV/cm]. The temperature of the apple cider samples was kept up to a maximum of 55 [°C] using a cooling system.

Alcohol content, pH, and phenolic compounds significantly alter the environment in which PEF treatments operate, varying the microbial membrane stability and the electrical properties of the beverage matrix. Alcohol can fluidize MLB and reduce membrane thickness, theoretically reducing the critical transmembrane potential required for electroporation. On the other hand, it may decrease the electrical conductivity and vary the temperature increase due to the ohmic heating. Analogously, acidic pH conditions have destabilizing effects on microbial membranes and cell wall structures. Additionally, they also influence ion mobility and charge screening, leading to modifications of the local electric field distribution. Phenolic compounds interact with membrane proteins and lipids, inducing oxidative stress responses that may affect the PEF-induced damage depending on concentration and exposure history. Current approaches often consider these effects by experimentally calibrating parameters for each beverage formulation, limiting the predictability and complicating microbial risk assessment for different products. Numerical models that account for alcohol concentration, pH-dependent conductivity, dielectric permittivity, and the stability of mechanical membranes can significantly improve the understanding of PEF treatment mechanisms in beverages. Numerical simulations can detect how local variations in conductivity and permittivity alter the electric and temperature field distributions inside the treatment chamber. Multiphysics simulations may offer a powerful tool to quantify and understand matrix–process interactions, enabling the virtual screening of process parameters to identify operating conditions that ensure sufficient microbial inactivation under conditions of high alcohol content and low pH. Additionally, numerical simulations could reinforce microbial safety assessments by connecting beverage compositions to spatially and temporally resolved inactivation distributions. This would be relevant for beverages with non-homogeneous composition and variable phenolic content, where local shielding or enhancement of the electric fields may occur.

## 9. Conclusions

PEF is a very promising non-thermal preservation technique that aims at replacing partially or completely traditional thermal treatment methods. PEF treatment is utilized to inactivate microorganisms in food, lowering enzymatic activity, and extending the shelf-life of food without excessively compromising the quality of the final product. When food, either in the solid or liquid state, undergoes a high-PEF treatment, it experiences pore formations and enlargement in cell membranes that leads to the inactivation of pathogenic microorganisms. In this manuscript, we presented the main existing mathematical and numerical models utilized in PEF processing according to their physical background, scale, complexity, and suitability for coupled multiphysics simulations. We have reviewed first the basic principles of the PEF process and the components of PEF devices in detail, including different types of treatment chambers among others. Subsequently, we have summarized the main electroporation models available in the literature, since those provide basic understandings for developing kinetic inactivation models. The latter have immediate practical applications because they predict the evolution of the microbial population under the action of PEF and represent also adequate tools to understand the effects of different processing parameters. Afterward, we reviewed the works related to numerical simulations of food processing using PEF. Numerical simulations provide detailed information concerning the fluid flow, electrical, thermal, and inactivation of the kinetic field, which are very difficult or nearly impossible to obtain experimentally. Those information are used to improve and optimize the PEF process, like, for instance, to optimize the shape of the chamber to improve the uniformity of the electric field and to avoid temperature inhomogeneities. Finally, we listed some applications of the PEF in food processing to demonstrate its efficacy concerning the inactivation of microorganisms. Although the PEF technique indicates encouraging results concerning the inactivation of microorganisms, PEF treatment processes need to be investigated further and improved to reach an equivalent or higher lethality than the current heat pasteurization treatments.

## Data Availability

Not applicable. This article is a review and does not contain any original data. No datasets were generated or analyzed during the current study.
